# Supplementary material for: Comparison of socio-economic determinants of COVID-19 testing and positivity in Canada: A multi-provincial analysis
Source: PLoS One. 2023 Aug 23;18(8):e0289292. doi: 10.1371/journal.pone.0289292 (PMC10446177; doi:10.1371/journal.pone.0289292)
Supplement: S1 Table — (DOCX) [file pone.0289292.s001.docx]

**S1 Supplemental Table 1:** Database data sources for the provinces of New Brunswick (NB), Manitoba (MB) and Ontario (ON).

| **Data Type** | | **Source Database** | | |
| --- | --- | --- | --- | --- |
| **Study cohort** | | NB | MB | ON |
|  | Population Selection | Citizen Registry | Manitoba Health Insurance Registry | OHIP |
|  | COVID-19 Testing and Results | COVID-19 Case Data | Manitoba Health COVID-19 Lab Testing and Results | OLIS |
|  | Demographics  (age, sex, place of residence, income, immigration status) | Citizen Registry | Manitoba Health Insurance Registry | OCR, CIHI-DAD, NACRS, OHIP, RPDB |
| **Health care usage** | | | | |
|  | Hospitalizations | CIHI-DAD | Manitoba Health Hospital Abstracts | CIHI-DAD |
|  | Physician claims | New Brunswick Physician Billing | Manitoba Health Medical Claims/Medical Services | OHIP |
|  | Comorbidities | CCDSS | Hospital Abstracts, Drug Program Information Network, Medical Claims/Medical Services | DAD, OHIP, ODB, and NACRS. |
| **Social determinants of health** | | | | |
|  | CIMD | Statistics Canada | Statistics Canada | Statistics Canada |
| **Supplemental** | |  |  |  |
|  | Air Quality | Canadian Urban Environment (CANUE) | Canadian Urban Environment (CANUE) | Canadian Urban Environment (CANUE) |
