# Supplementary material for: Comparison of socio-economic determinants of COVID-19 testing and positivity in Canada: A multi-provincial analysis
Source: PLoS One. 2023 Aug 23;18(8):e0289292. doi: 10.1371/journal.pone.0289292 (PMC10446177; doi:10.1371/journal.pone.0289292)
Supplement: S2 Table — (DOCX) [file pone.0289292.s002.docx]

**S2 Supplemental Table 2:** Odds ratios and confidence intervals for multivariable regression models of the odds of being tested for SARS-CoV-2, in New Brunswick (NB), Manitoba (MB), and Ontario (ON), including additional covariates for comorbidities and air pollution.

| **Variable** | **NB** | **MB** | **ON** |
| --- | --- | --- | --- |
| Age group: 5-19 vs 0-4 | **0.78 (0.75, 0.80)** | **1.37 (1.34, 1.40)** | **1.05 (1.05, 1.05)** |
| Age group: 20-34 vs 0-4 | **0.89 (0.86, 0.91)** | **1.94 (1.91, 1.98)** | **1.48 (1.48, 1.49)** |
| Age group: 35-49 vs 0-4 | **0.83 (0.81, 0.86)** | **1.78 (1.75, 1.82)** | **1.27 (1.27, 1.27)** |
| Age group: 50-64 vs 0-4 | **0.61 (0.59, 0.62)** | **1.32 (1.29, 1.34)** | **1.07 (1.07, 1.08)** |
| Age group: 65-74 vs 0-4 | **0.42 (0.41, 0.44)** | **0.93 (0.91, 0.95)** | **0.76 (0.76, 0.76)** |
| Age group: 75-84 vs 0-4 | **0.38 (0.36, 0.39)** | **0.86 (0.84, 0.89)** | **0.69 (0.69, 0.69)** |
| Age group: 85+ vs 0-4 | **0.40 (0.38, 0.42)** | **0.89 (0.86, 0.93)** | **0.94 (0.94, 0.95)** |
| Sex: Female vs Male | **1.28 (1.27, 1.30)** | **1.21 (1.20, 1.22)** | **1.09 (1.09, 1.09)** |
| Income quintile: 2 vs 1 | 1.02 (1.00, 1.04) | **0.90 (0.89, 0.92)** | **0.94 (0.94, 0.95)** |
| Income quintile: 3 vs 1 | **1.08 (1.06, 1.11)** | **0.88 (0.87, 0.90)** | **0.99 (0.99, 1.00)** |
| Income quintile: 4 vs 1 | **1.20 (1.17, 1.23)** | **0.90 (0.88, 0.91)** | **1.04 (1.04, 1.04)** |
| Income quintile: 5 vs 1 | **1.38 (1.34, 1.42)** | **0.88 (0.87, 0.90)** | **1.10 (1.10, 1.10)** |
| Rurality: Urban vs Rural | **1.45 (1.43, 1.47)** | **1.03 (1.02, 1.05)** | **1.06 (1.06, 1.06)** |
| Hospital admissions: 1 vs 0 | **1.37 (1.35, 1.39)** | **1.39 (1.37, 1.41)** | **0.87 (0.86, 0.87)** |
| Hospital admissions: 2 vs 0 | **1.52 (1.49, 1.56)** | **1.63 (1.59, 1.66)** | **1.06 (1.05, 1.07)** |
| Hospital admissions: ≥3 vs 0 | **1.87 (1.82, 1.91)** | **2.30 (2.23, 2.37)** | **1.57 (1.56, 1.58)** |
| Comorbidities: COPD | **1.34 (1.31, 1.37)** | **1.36 (1.28, 1.43)** | **1.27 (1.25, 1.28)** |
| Comorbidities: hypertension | **1.08 (1.06, 1.09)** | **1.10 (1.09, 1.11)** | **1.08 (1.08, 1.09)** |
| Comorbidities: diabetes | **1.11 (1.09, 1.13)** | **1.29 (1.27, 1.31)** | **1.08 (1.07, 1.08)** |
| Comorbidities: cancer | **1.03 (1.00, 1.06)** | N/A | **1.31 (1.30, 1.32)** |
| Comorbidities: asthma | N/A | **1.44 (1.41, 1.46)** | **1.23 (1.23, 1.24)** |
| Comorbidities: heart disease | N/A | **1.23 (1.19, 1.26)** | **1.18 (1.17, 1.19)** |
| Comorbidities: dementia/frailty | N/A | **1.32 (1.25, 1.40)** | **1.80 (1.78, 1.82)** |
| Air pollution - PM_2.5_ category (µg/m^3^ per year): 6 to < 7 vs 2 to <6 | N/A | **0.98 (0.97, 1.00)** | **1.05 (1.05, 1.06)** |
| Air pollution - PM_2.5_ (µg/m^3^ per year): 7 to < 8 vs 2 to <6 | N/A | **1.04 (1.03, 1.06)** | **0.96 (0.96, 0.97)** |
| Air pollution - PM_2.5_ category (µg/m^3^ per year): 8 to < 9 vs 2 to <6 | N/A | **1.04 (1.02, 1.07)** | **1.02 (1.02, 1.03)** |
| Air pollution - PM_2.5_ category (µg/m^3^ per year): ≥9 vs 2 to <6 | N/A | N/A | **0.91 (0.91, 0.92)** |
| Air pollution: NO_2_ category (ppb per year): 6 to 8 vs 0 to 6 | N/A | **1.08 (1.06, 1.09)** | **0.97 (0.97, 0.97)** |
| Air pollution: NO_2_ category (ppb per year): ≥ 8 vs 0 to 6 | N/A | **1.11 (1.09, 1.13)** | **1.06 (1.06, 1.07)** |
| CIMD Residential instability: 2 vs 1 | **1.04 (1.03, 1.06)** | **0.90 (0.88, 0.91)** | **0.97 (0.97, 0.97)** |
| CIMD Residential instability: 3 vs 1 | **1.15 (1.13, 1.17)** | **0.92 (0.91, 0.94)** | **1.02 (1.02, 1.02)** |
| CIMD Residential instability: 4 vs 1 | **1.23 (1.20, 1.25)** | **0.86 (0.85, 0.87)** | **1.06 (1.05, 1.06)** |
| CIMD Residential instability: 5 vs 1 | **1.11 (1.08, 1.14)** | **0.89 (0.88, 0.90)** | **1.02 (1.01, 1.02)** |
| CIMD Economic dependency: 2 vs 1 | 1.01 (0.99, 1.03) | **0.96 (0.94, 0.97)** | **1.01 (1.01, 1.01)** |
| CIMD Economic dependency: 3 vs 1 | 1.02 (0.99, 1.04) | **1.02 (1.01, 1.03)** | 1.00 (1.00, 1.00) |
| CIMD Economic dependency: 4 vs 1 | **1.08 (1.05, 1.10)** | **1.01 (0.99, 1.02)** | **1.00 (0.99, 1.00)** |
| CIMD Economic dependency: 5 vs 1 | **1.11 (1.09, 1.14)** | **0.88 (0.87, 0.89)** | 1.00 (1.00, 1.00) |
| CIMD Ethnocultural composition: 2 vs 1 | **0.95 (0.94, 0.97)** | **1.01 (0.99, 1.02)** | **1.03 (1.03, 1.03)** |
| CIMD Ethnocultural composition: 3 vs 1 | **0.93 (0.92, 0.96)** | **1.02 (1.01, 1.04)** | **1.04 (1.04, 1.05)** |
| CIMD Ethnocultural composition: 4 vs 1 | **0.90 (0.88, 0.93)** | **0.89 (0.87, 0.90)** | **1.01 (1.01, 1.01)** |
| CIMD Ethnocultural composition: 5 vs 1 | **0.77 (0.74, 0.81)** | **0.73 (0.72, 0.75)** | **0.89 (0.89, 0.90)** |
| CIMD Situational vulnerability: 2 vs 1 | **0.94 (0.93, 0.96)** | **0.98 (0.96, 0.99)** | **1.01 (1.00, 1.01)** |
| CIMD Situational vulnerability: 3 vs 1 | **1.05 (1.03, 1.08)** | **0.92 (0.91, 0.94)** | **1.01 (1.01, 1.01)** |
| CIMD Situational vulnerability: 4 vs 1 | **1.03 (1.01, 1.05)** | **0.93 (0.92, 0.95)** | **1.00 (1.00, 1.01)** |
| CIMD Situational vulnerability: 5 vs 1 | 1.02 (1.00, 1.05) | **1.04 (1.02, 1.06)** | **0.99 (0.99, 1.00)** |

Adjusted odds ratios and 95% CIs are presented. The adjusted model contains all variables listed in the table and does not contain additional covariates that are not listed in the table.
