# Supplementary material for: Comparison of socio-economic determinants of COVID-19 testing and positivity in Canada: A multi-provincial analysis
Source: PLoS One. 2023 Aug 23;18(8):e0289292. doi: 10.1371/journal.pone.0289292 (PMC10446177; doi:10.1371/journal.pone.0289292)
Supplement: S3 Table — (DOCX) [file pone.0289292.s003.docx]

**S3 Supplemental Table 3:** Odds ratios and confidence intervals for multivariable regression models of the odds of testing positive for SARS-CoV-2 among tested individuals, in New Brunswick (NB), Manitoba (MB), and Ontario (ON), including additional covariates for comorbidities and air pollution.

| **Variable** | **NB** | **MB** | **ON** |
| --- | --- | --- | --- |
| Age group: 5-19 vs 0-4 | **1.42 (1.02, 1.96)** | **1.34 (1.26, 1.43)** | **1.57 (1.53, 1.61)** |
| Age group: 20-34 vs 0-4 | **1.71 (1.25, 2.34)** | **1.29 (1.21, 1.37)** | **1.46 (1.42, 1.50)** |
| Age group: 35-49 vs 0-4 | **1.81 (1.33, 2.47)** | **1.16 (1.09, 1.24)** | **1.52 (1.48, 1.56)** |
| Age group: 50-64 vs 0-4 | **2.23 (1.64, 3.05)** | **1.20 (1.12, 1.28)** | **1.53 (1.49, 1.57)** |
| Age group: 65-74 vs 0-4 | **1.77 (1.25, 2.50)** | 0.93 (0.86, 1.00) | **1.22 (1.19, 1.26)** |
| Age group: 75-84 vs 0-4 | **2.20 (1.51, 3.22)** | **0.89 (0.81, 0.98)** | **1.10 (1.06, 1.14)** |
| Age group: 85+ vs 0-4 | **4.51 (3.06, 6.65)** | 1.04 (0.93, 1.18) | 0.96 (0.91, 1.00) |
| Sex: Female vs Male | **0.90 (0.81, 0.99)** | **0.88 (0.86, 0.90)** | **0.81 (0.80, 0.81)** |
| Income quintile: 2 vs 1 | 0.96 (0.81, 1.14) | **0.68 (0.66, 0.71)** | **0.82 (0.80, 0.83)** |
| Income quintile: 3 vs 1 | 1.10 (0.91, 1.33) | **0.58 (0.56, 0.61)** | **0.74 (0.73, 0.75)** |
| Income quintile: 4 vs 1 | 1.04 (0.84, 1.28) | **0.56 (0.53, 0.59)** | **0.62 (0.60, 0.63)** |
| Income quintile: 5 vs 1 | 1.24 (0.99, 1.65) | **0.45 (0.43, 0.48)** | **0.53 (0.51, 0.54)** |
| Rurality: Urban vs Rural | **1.46 (1.29, 1.65)** | **0.85 (0.81, 0.89)** | **1.08 (1.05, 1.11)** |
| Hospital admissions: 1 vs 0 | **0.77 (0.66, 0.90)** | **0.95 (0.92, 0.99)** | **0.91 (0.90, 0.93)** |
| Hospital admissions: 2 vs 0 | **0.73 (0.59, 0.89)** | 0.94 (0.88, 1.00) | **0.79 (0.76, 0.81)** |
| Hospital admissions: ≥3 vs 0 | 0.84 (0.68, 1.04) | 1.00 (0.92, 1.07) | **0.55 (0.53, 0.58)** |
| Comorbidities: COPD | **0.82 (0.68, 1.00)** | 1.02 (0.88, 1.18) | **0.71 (0.68, 0.73)** |
| Comorbidities: hypertension | 1.06 (0.92, 1.22) | **1.09 (1.05, 1.14)** | **1.08 (1.07, 1.10)** |
| Comorbidities: diabetes | 1.12 (0.95, 1.31) | **1.29 (1.24, 1.35)** | **1.32 (1.30, 1.34)** |
| Comorbidities: cancer | 0.88 (0.70, 1.11) | N/A | **0.70 (0.68, 0.73)** |
| Comorbidities: asthma | N/A | **0.77 (0.72, 0.81)** | **0.87 (0.86, 0.88)** |
| Comorbidities: heart disease | N/A | 0.97 (0.90, 1.05) | **0.88 (0.85, 0.90)** |
| Comorbidities: dementia/frailty | N/A | **1.58 (1.38, 1.81)** | **0.90 (0.86, 0.94)** |
| Air pollution - PM_2.5_ category (µg/m^3^ per year): 6 to < 7 vs 2 to <6 | N/A | **1.15 (1.10, 1.19)** | **1.40 (1.37, 1.43)** |
| Air pollution - PM_2.5_ (µg/m^3^ per year): 7 to < 8 vs 2 to <6 | N/A | **1.11 (1.05, 1.17)** | **1.82 (1.78, 1.86)** |
| Air pollution - PM_2.5_ category (µg/m^3^ per year): 8 to < 9 vs 2 to <6 | N/A | **1.13 (1.06, 1.21)** | **1.82 (1.78, 1.86)** |
| Air pollution - PM_2.5_ category (µg/m^3^ per year): ≥9 vs 2 to <6 | N/A | N/A | **1.86 (1.82, 1.91)** |
| Air pollution: NO_2_ category (ppb per year): 6 to 8 vs 0 to 6 | N/A | **0.71 (0.68, 0.75)** | **1.14 (1.13, 1.16)** |
| Air pollution: NO_2_ category (ppb per year): ≥ 8 vs 0 to 6 | N/A | **0.81 (0.77, 0.86)** | **1.24 (1.22, 1.26)** |
| CIMD Residential instability: 2 vs 1 | 0.91 (0.78, 1.07) | **0.74 (0.71, 0.77)** | **0.95 (0.93, 0.96)** |
| CIMD Residential instability: 3 vs 1 | 1.09 (0.93, 1.28) | **0.68 (0.65, 0.71)** | **0.86 (0.84, 0.87)** |
| CIMD Residential instability: 4 vs 1 | 1.06 (0.89, 1.28) | **0.60 (0.57, 0.62)** | **0.67 (0.66, 0.68)** |
| CIMD Residential instability: 5 vs 1 | 0.98 (0.78, 1.24) | **0.54 (0.52, 0.57)** | **0.60 (0.59, 0.61)** |
| CIMD Economic dependency: 2 vs 1 | **1.60 (1.25, 2.05)** | **0.93 (0.89, 0.97)** | **1.02 (1.01, 1.03)** |
| CIMD Economic dependency: 3 vs 1 | **1.91 (1.51, 2.42)** | 1.04 (1.00, 1.08) | **0.95 (0.94, 0.96)** |
| CIMD Economic dependency: 4 vs 1 | **2.55 (2.02, 3.23)** | **1.08 (1.04, 1.13)** | **0.95 (0.94, 0.97)** |
| CIMD Economic dependency: 5 vs 1 | **2.04 (1.60, 2.60)** | **0.88 (0.84, 0.92)** | **0.88 (0.87, 0.90)** |
| CIMD Ethnocultural composition: 2 vs 1 | **0.85 (0.76, 0.95)** | **1.13 (1.08, 1.19)** | **1.07 (1.04, 1.10)** |
| CIMD Ethnocultural composition: 3 vs 1 | 1.11 (0.94, 1.30) | **1.45 (1.38, 1.52)** | **1.30 (1.26, 1.33)** |
| CIMD Ethnocultural composition: 4 vs 1 | 1.01 (0.80, 1.28) | **1.50 (1.43, 1.59)** | **1.75 (1.70, 1.80)** |
| CIMD Ethnocultural composition: 5 vs 1 | 1.15 (0.80, 1.65) | **1.71 (1.61, 1.80)** | **3.02 (2.94, 3.12)** |
| CIMD Situational vulnerability: 2 vs 1 | 0.85 (0.69, 1.04) | 1.03 (0.98, 1.09) | **1.15 (1.13, 1.16)** |
| CIMD Situational vulnerability: 3 vs 1 | **1.29 (1.05, 1.58)** | **1.19 (1.12, 1.25)** | **1.19 (1.17, 1.20)** |
| CIMD Situational vulnerability: 4 vs 1 | **1.60 (1.32, 1.95)** | **1.22 (1.15, 1.29)** | **1.19 (1.17, 1.21)** |
| CIMD Situational vulnerability: 5 vs 1 | **1.29 (1.04, 1.61)** | **1.36 (1.28, 1.45)** | **1.36 (1.33, 1.38)** |

Adjusted odds ratios and 95% CIs are presented. The adjusted model contains all variables listed in the table and does not contain additional covariates that are not listed in the table.
