# Supplementary material for: Comparison of socio-economic determinants of COVID-19 testing and positivity in Canada: A multi-provincial analysis
Source: PLoS One. 2023 Aug 23;18(8):e0289292. doi: 10.1371/journal.pone.0289292 (PMC10446177; doi:10.1371/journal.pone.0289292)
Supplement: S4 Table — Bolded values indicate significance. (DOCX) [file pone.0289292.s004.docx]

**S4 Supplemental Table 4:** Odds ratios and confidence limits for univariate regression models of SARS-CoV-2 tested individuals in New Brunswick (NB), Manitoba (MB), and Ontario (ON). Bolded values indicate significance.

| **Variable** | **NB** | **MB** | **ON** |
| --- | --- | --- | --- |
| Age group: 5-19 vs 0-4 | **0.79 (0.77, 0.82)** | **1.14 (1.12, 1.17)** | **1.02 (1.01, 1.02)** |
| Age group: 20-34 vs 0-4 | **0.94 (0.91, 0.97)** | **1.70 (1.67, 1.73)** | **1.48 (1.48, 1.49)** |
| Age group: 35-49 vs 0-4 | **0.91 (0.89, 0.94)** | **1.57 (1.54, 1.60)** | **1.27 (1.26, 1.28)** |
| Age group: 50-64 vs 0-4 | **0.70 (0.68, 0.72)** | **1.24 (1.21, 1.26)** | **1.13 (1.12, 1.13)** |
| Age group: 65-74 vs 0-4 | **0.55 (0.53, 0.57)** | 0.98 (0.96, 1.00) | **0.89 (0.88, 0.89)** |
| Age group: 75-84 vs 0-4 | **0.55 (0.53, 0.57)** | **1.10 (1.07, 1.13)** | **0.91 (0.90, 0.91)** |
| Age group: 85+ vs 0-4 | **0.63 (0.60, 0.66)** | **1.66 (1.62, 1.71)** | **1.43 (1.41, 1.44)** |
| Sex: Female vs Male | **1.30 (1.29, 1.31)** | **1.23 (1.22, 1.24)** | **1.19 (1.19, 1.19)** |
| Income quintile: 2 vs 1 | 1.00 (0.98, 1.02) | **0.86 (0.85, 0.87)** | 1.00 (1.00, 1.01) |
| Income quintile: 3 vs 1 | 1.02 (1.00, 1.03) | **0.83 (0.82, 0.84)** | **1.02 (1.02, 1.03)** |
| Income quintile: 4 vs 1 | **1.08 (1.06, 1.10)** | **0.82 (0.81, 0.83)** | **1.04 (1.04, 1.05)** |
| Income quintile: 5 vs 1 | **1.18 (1.16, 1.20)** | **0.89 (0.88, 0.90)** | **1.09 (1.09, 1.10)** |
| Rurality: Urban vs Rural | **1.37 (1.35, 1.38)** | **1.03 (1.02, 1.04)** | **1.09 (1.08, 1.09)** |
| Hospital admissions: 1 vs 0 | **1.26 (1.24, 1.28)** | **1.34 (1.33, 1.36)** | **1.24 (1.23, 1.24)** |
| Hospital admissions: 2 vs 0 | **1.42 (1.39, 1.45)** | **1.79 (1.75, 1.82)** | **1.59 (1.58, 1.60)** |
| Hospital admissions: ≥3 vs 0 | **1.63 (1.59, 1.67)** | **2.71 (2.64, 2.78)** | **2.63 (2.61, 2.66)** |
| Comorbidities: COPD | **1.08 (1.06, 1.11)** | **2.28 (2.17, 2.39)** | **1.36 (1.35, 1.37)** |
| Comorbidities: hypertension | **0.85 (0.84, 0.86)** | **1.04 (1.03, 1.05)** | **0.97 (0.97, 0.97)** |
| Comorbidities: diabetes | **0.95 (0.93, 0.97)** | **1.29 (1.28, 1.31)** | **1.02 (1.02, 1.02)** |
| Comorbidities: cancer | **0.92 (0.89, 0.94)** | **N/A** | **1.37 (1.36, 1.38)** |
| Comorbidities: asthma | **N/A** | **1.50 (1.47, 1.52)** | **1.31 (1.31, 1.31)** |
| Comorbidities: heart disease | **N/A** | **1.53 (1.50, 1.56)** | **1.23 (1.22, 1.24)** |
| Comorbidities: dementia/frailty | **N/A** | **2.63 (2.54, 2.72)** | **2.00 (1.98, 2.02)** |
| Air pollution - PM_2.5_ category (µg/m^3^ per year): 6 to < 7 vs 2 to <6 | **N/A** | **0.98 (0.97, 0.99)** | **1.02 (1.02, 1.03)** |
| Air pollution - PM_2.5_ (µg/m^3^ per year): 7 to < 8 vs 2 to <6 | **N/A** | **1.08 (1.07, 1.09)** | **0.93 (0.92, 0.93)** |
| Air pollution - PM_2.5_ category (µg/m^3^ per year): 8 to < 9 vs 2 to <6 | **N/A** | **1.10 (1.08, 1.12)** | **1.01 (1.01, 1.02)** |
| Air pollution - PM_2.5_ category (µg/m^3^ per year): ≥9 vs 2 to <6 | **N/A** | **N/A** | **0.91 (0.91, 0.92)** |
| Air pollution: NO_2_ category (ppb per year): 6 to 8 vs 0 to 6 | **N/A** | 1.00 (0.99, 1.01) | **0.96 (0.96, 0.97)** |
| Air pollution: NO_2_ category (ppb per year): ≥ 8 vs 0 to 6 | **N/A** | **1.06 (1.05, 1.07)** | **0.99 (0.99, 1.00)** |
| CIMD Residential instability: 2 vs 1 | **0.93 (0.91, 0.94)** | **0.92 (0.91, 0.93)** | **0.99 (0.99, 1.00)** |
| CIMD Residential instability: 3 vs 1 | **0.98 (0.97, 1.00)** | **0.96 (0.94, 0.97)** | **1.03 (1.02, 1.03)** |
| CIMD Residential instability: 4 vs 1 | **1.04 (1.02, 1.06)** | **0.87 (0.86, 0.88)** | **1.06 (1.05, 1.06)** |
| CIMD Residential instability: 5 vs 1 | **0.97 (0.95, 0.99)** | **0.97 (0.96, 0.98)** | **1.01 (1.01, 1.02)** |
| CIMD Economic dependency: 2 vs 1 | **0.93 (0.92, 0.95)** | 1.00 (0.99, 1.01) | **0.98 (0.98, 0.99)** |
| CIMD Economic dependency: 3 vs 1 | **0.89 (0.87, 0.91)** | **1.02 (1.01, 1.03)** | **0.97 (0.97, 0.97)** |
| CIMD Economic dependency: 4 vs 1 | **0.88 (0.87, 0.90)** | **1.02 (1.01, 1.03)** | **0.95 (0.95, 0.96)** |
| CIMD Economic dependency: 5 vs 1 | **0.84 (0.82, 0.85)** | **0.87 (0.86, 0.88)** | **0.94 (0.94, 0.95)** |
| CIMD Ethnocultural composition: 2 vs 1 | **0.97 (0.96, 0.98)** | **1.10 (1.08, 1.11)** | **1.01 (1.01, 1.02)** |
| CIMD Ethnocultural composition: 3 vs 1 | **1.04 (1.02, 1.06)** | **1.15 (1.14, 1.17)** | **1.06 (1.06, 1.07)** |
| CIMD Ethnocultural composition: 4 vs 1 | **1.05 (1.02, 1.07)** | **1.04 (1.03, 1.06)** | **1.06 (1.05, 1.06)** |
| CIMD Ethnocultural composition: 5 vs 1 | **0.88 (0.85, 0.92)** | **0.96 (0.95, 0.97)** | **0.95 (0.94, 0.95)** |
| CIMD Situational vulnerability: 2 vs 1 | **0.87 (0.86, 0.89)** | **0.95 (0.94, 0.96)** | **0.99 (0.99, 1.00)** |
| CIMD Situational vulnerability: 3 vs 1 | **0.90 (0.88, 0.92)** | **0.91 (0.90, 0.92)** | **0.98 (0.98, 0.98)** |
| CIMD Situational vulnerability: 4 vs 1 | **0.84 (0.83, 0.86)** | **0.93 (0.92, 0.95)** | **0.97 (0.97, 0.97)** |
| CIMD Situational vulnerability: 5 vs 1 | **0.77 (0.76, 0.78)** | **1.12 (1.10, 1.13)** | **0.96 (0.96, 0.96)** |

*CIMD is the Canadian Index of Multiple Deprivation. It is scored from 1 to 5, with 1 being the least marginalized and 5 being the most marginalized.
