# Supplementary material for: Comparison of socio-economic determinants of COVID-19 testing and positivity in Canada: A multi-provincial analysis
Source: PLoS One. 2023 Aug 23;18(8):e0289292. doi: 10.1371/journal.pone.0289292 (PMC10446177; doi:10.1371/journal.pone.0289292)
Supplement: S5 Table — Bolded values indicate significance. (DOCX) [file pone.0289292.s005.docx]

**S5 Supplemental Table 5:**  Odds ratios and confidence limits for univariate regression models of individuals testing positive for SARS-CoV-2 in New Brunswick (NB), Manitoba (MB), and Ontario (ON). Bolded values indicate significance.

| **Variable** | **NB** | **MB** | **ON** |
| --- | --- | --- | --- |
| Age group: 5-19 vs 0-4 | **1.41 (1.02, 1.95)** | **1.28 (1.20, 1.35)** | **1.62 (1.58, 1.66)** |
| Age group: 20-34 vs 0-4 | **1.72 (1.26, 2.35)** | **1.21 (1.14, 1.28)** | **1.62 (1.58, 1.66)** |
| Age group: 35-49 vs 0-4 | **1.80 (1.32, 2.45)** | **1.06 (1.01, 1.13)** | **1.69 (1.65, 1.73)** |
| Age group: 50-64 vs 0-4 | **2.31 (1.70, 3.13)** | **1.13 (1.07, 1.20)** | **1.67 (1.63, 1.71)** |
| Age group: 65-74 vs 0-4 | **1.79 (1.29, 2.49)** | **0.93 (0.87, 1.00)** | **1.21 (1.18, 1.24)** |
| Age group: 75-84 vs 0-4 | **2.17 (1.52, 3.09)** | 1.07 (0.99, 1.15) | 1.03 (1.00, 1.06) |
| Age group: 85+ vs 0-4 | **4.38 (3.06, 6.27)** | **1.71 (1.59, 1.85)** | **0.84 (0.81, 0.87)** |
| Sex: Female vs Male | **0.89 (0.81, 0.99)** | **0.88 (0.86, 0.90)** | **0.79 (0.78, 0.79)** |
| Income quintile: 2 vs 1 | 0.96 (0.83, 1.11) | **0.57 (0.55, 0.58)** | **0.85 (0.84, 0.86)** |
| Income quintile: 3 vs 1 | 0.93 (0.80, 1.08) | **0.47 (0.45, 0.48)** | **0.80 (0.79, 0.81)** |
| Income quintile: 4 vs 1 | **0.78 (0.67, 0.91)** | **0.43 (0.42, 0.45)** | **0.62 (0.62, 0.63)** |
| Income quintile: 5 vs 1 | **0.79 (0.68, 0.91)** | **0.35 (0.34, 0.36)** | **0.47 (0.46, 0.47)** |
| Rurality: Urban vs Rural | **1.15 (1.03, 1.28)** | **0.77 (0.75, 0.79)** | **2.90 (2.84, 2.96)** |
| Hospital admissions: 1 vs 0 | **0.85 (0.73, 0.99)** | 1.02 (0.99, 1.06) | **0.76 (0.75, 0.77)** |
| Hospital admissions: 2 vs 0 | **0.81 (0.67, 0.99)** | **1.15 (1.08, 1.21)** | **0.63 (0.61, 0.65)** |
| Hospital admissions: ≥3 vs 0 | 1.01 (0.83, 1.23) | **1.39 (1.31, 1.47)** | **0.44 (0.42, 0.46)** |
| Comorbidities: COPD | 1.06 (0.89, 1.27) | **1.20 (1.07, 1.35)** | **0.48 (0.46, 0.50)** |
| Comorbidities: hypertension | **1.34 (1.20, 1.49)** | **1.10 (1.07, 1.14)** | **0.97 (0.96, 0.98)** |
| Comorbidities: diabetes | **1.32 (1.14, 1.53)** | **1.44 (1.39, 1.49)** | **1.28 (1.27, 1.30)** |
| Comorbidities: cancer | 1.08 (0.87, 1.35) | **N/A** | **0.55 (0.53, 0.58)** |
| Comorbidities: asthma | **N/A** | **0.76 (0.72, 0.80)** | **0.85 (0.84, 0.86)** |
| Comorbidities: heart disease | **N/A** | **1.26 (1.19, 1.33)** | **0.69 (0.67, 0.70)** |
| Comorbidities: dementia/frailty | **N/A** | **2.20 (2.06, 2.35)** | **0.57 (0.55, 0.60)** |
| Air pollution - PM_2.5_ category (µg/m^3^ per year): 6 to < 7 vs 2 to <6 | **N/A** | 1.01 (0.98, 1.04) | **1.74 (1.70, 1.78)** |
| Air pollution - PM_2.5_ (µg/m^3^ per year): 7 to < 8 vs 2 to <6 | **N/A** | **0.96 (0.93, 0.98)** | **3.93 (3.87, 4.00)** |
| Air pollution - PM_2.5_ category (µg/m^3^ per year): 8 to < 9 vs 2 to <6 | **N/A** | **1.15 (1.10, 1.20)** | **3.87 (3.80, 3.93)** |
| Air pollution - PM_2.5_ category (µg/m^3^ per year): ≥9 vs 2 to <6 | **N/A** | **N/A** | **2.78 (2.72, 2.84)** |
| Air pollution: NO_2_ category (ppb per year): 6 to 8 vs 0 to 6 | **N/A** | **0.62 (0.60, 0.64)** | **2.12 (2.10, 2.15)** |
| Air pollution: NO_2_ category (ppb per year): ≥ 8 vs 0 to 6 | **N/A** | **0.91 (0.89, 0.93)** | **3.41 (3.37, 3.45)** |
| CIMD Residential instability: 2 vs 1 | 1.00 (0.86, 1.16) | **0.52 (0.51, 0.54)** | **1.15 (1.14, 1.17)** |
| CIMD Residential instability: 3 vs 1 | 1.12 (0.96, 1.30) | **0.54 (0.52, 0.56)** | **1.18 (1.17, 1.20)** |
| CIMD Residential instability: 4 vs 1 | **1.20 (1.02, 1.40)** | **0.52 (0.50, 0.54)** | **1.07 (1.06, 1.08)** |
| CIMD Residential instability: 5 vs 1 | **1.21 (1.02, 1.42)** | **0.64 (0.63, 0.66)** | **1.40 (1.38, 1.41)** |
| CIMD Economic dependency: 2 vs 1 | **1.61 (1.27, 2.04)** | **0.89 (0.86, 0.92)** | **1.19 (1.17, 1.20)** |
| CIMD Economic dependency: 3 vs 1 | **2.06 (1.66, 2.57)** | 0.97 (0.94, 1.00) | **1.08 (1.07, 1.09)** |
| CIMD Economic dependency: 4 vs 1 | **2.73 (2.21, 3.36)** | 0.98 (0.95, 1.02) | **1.09 (1.08, 1.10)** |
| CIMD Economic dependency: 5 vs 1 | **2.19 (1.78, 2.70)** | **0.67 (0.64, 0.69)** | **0.79 (0.78, 0.81)** |
| CIMD Ethnocultural composition: 2 vs 1 | **0.79 (0.71, 0.89)** | **1.15 (1.10, 1.20)** | **1.18 (1.15, 1.22)** |
| CIMD Ethnocultural composition: 3 vs 1 | 0.91 (0.78, 1.05) | **1.23 (1.18, 1.28)** | **1.62 (1.58, 1.67)** |
| CIMD Ethnocultural composition: 4 vs 1 | 0.90 (0.72, 1.13) | **1.20 (1.15, 1.25)** | **2.46 (2.40, 2.52)** |
| CIMD Ethnocultural composition: 5 vs 1 | 1.02 (0.73, 1.44) | **1.58 (1.52, 1.64)** | **5.53 (5.40, 5.67)** |
| CIMD Situational vulnerability: 2 vs 1 | 0.92 (0.76, 1.11) | **1.09 (1.04, 1.14)** | **1.22 (1.21, 1.24)** |
| CIMD Situational vulnerability: 3 vs 1 | **1.45 (1.22, 1.73)** | **1.34 (1.28, 1.41)** | **1.28 (1.26, 1.29)** |
| CIMD Situational vulnerability: 4 vs 1 | **1.83 (1.57, 2.13)** | **1.41 (1.35, 1.47)** | **1.33 (1.31, 1.35)** |
| CIMD Situational vulnerability: 5 vs 1 | **1.34 (1.14, 1.58)** | **2.39 (2.30, 2.48)** | **1.54 (1.52, 1.56)** |

*CIMD is the Canadian Index of Multiple Deprivation. It is scored from 1 to 5, with 1 being the least marginalized and 5 being the most marginalized.
