# Supplementary figures and images for: Comparison of socio-economic determinants of COVID-19 testing and positivity in Canada: A multi-provincial analysis
Source: PLoS One. 2023 Aug 23;18(8):e0289292. doi: 10.1371/journal.pone.0289292 (PMC10446177; doi:10.1371/journal.pone.0289292)

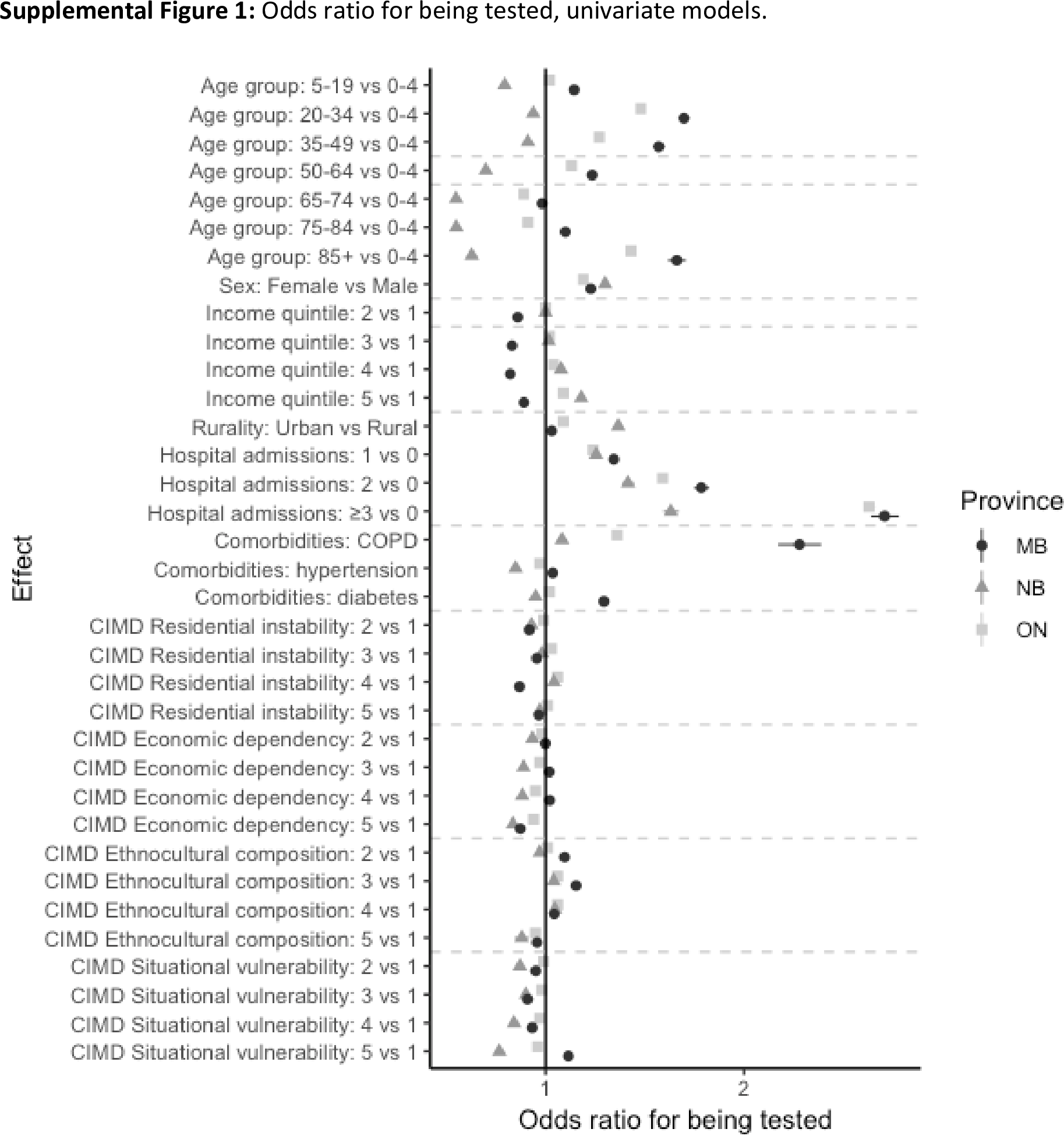

Supplement: S1 Fig — (TIF) [file pone.0289292.s006.tif]

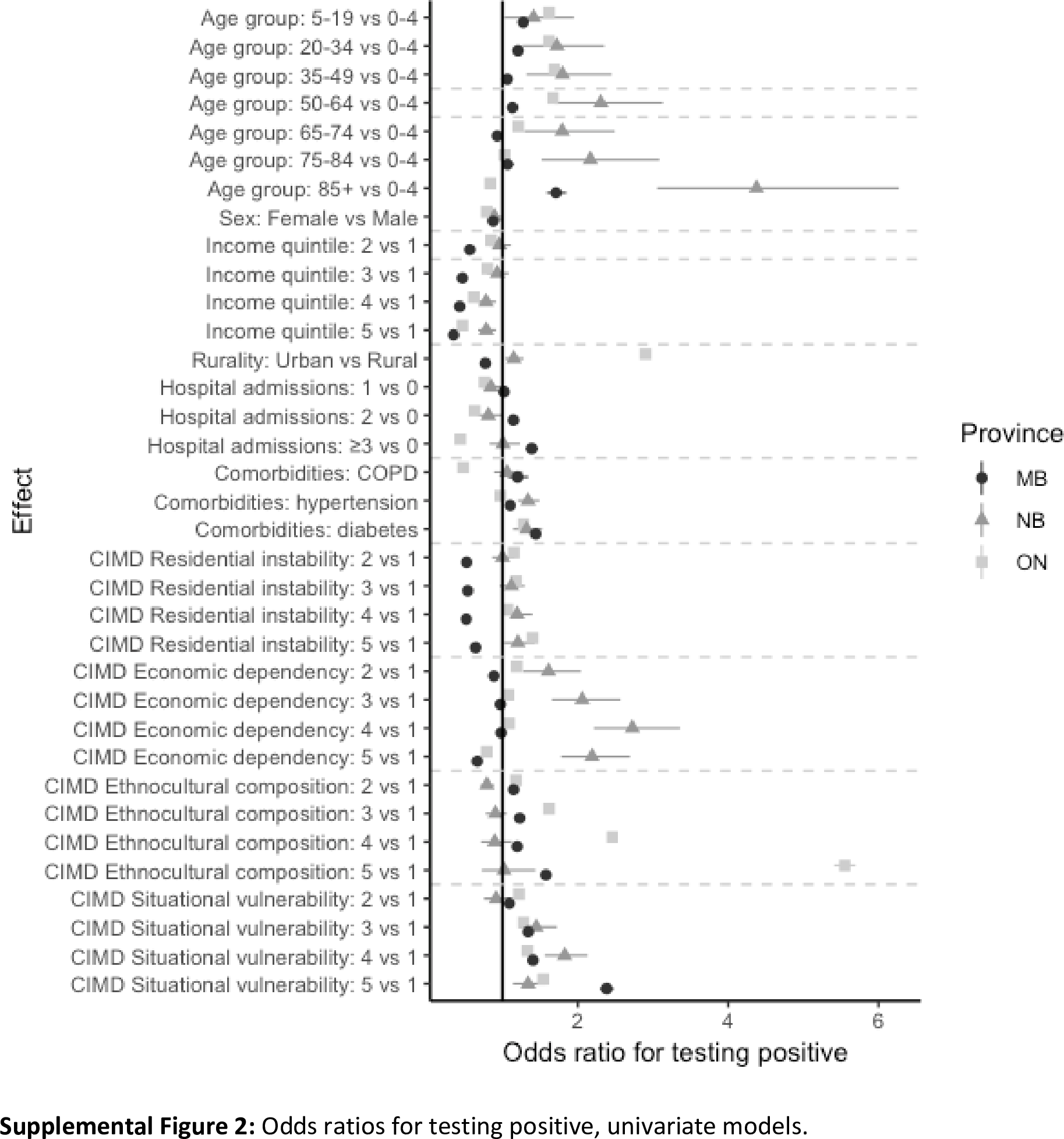

Supplement: S2 Fig — (TIF) [file pone.0289292.s007.tif]
