# Supplementary material for: Comparison of socio-economic determinants of COVID-19 testing and positivity in Canada: A multi-provincial analysis
Source: PLoS One. 2023 Aug 23;18(8):e0289292. doi: 10.1371/journal.pone.0289292 (PMC10446177; doi:10.1371/journal.pone.0289292)
Supplement: Attachment — Submitted filename: PONE-D-22-25812_reviewer1.docx [file pone.0289292.s008.docx]

Reviewer Response:

Thank you for your manuscript submission. Overall, this paper is interesting and provides some insights that are of great interest for those in public health to review and address in order to improve equity and access to care. These findings have the potential to make meaningful impacts for all outbreaks and general disease mitigation that go beyond COVID-19. That being said, I have several questions and concerns that resonate from the manuscript in its current form. These are listed below in bulleted form, with major and minor subheadings addressing them accordingly. Additional information and comments are located throughout in track changes.

Major Questions/Concerns:

- Regarding the analysis in general: it appears that the analysis was first conducted prior by each province? This is a bit confusing and would like some clarification on what was analyzed prior to your team receiving the data. Did you mean they collated and cleaned the data? Aside from that, the analysis seems a bit misleading in a few areas and potentially biased in another. Specifically, the data reported on comorbidities seems to be inconsistent across province. Some provinces contain all or some, but very few contain all comorbidities. A true comparison would be difficult to assess without addressing major potential biases.
- The analysis was reported to be a “multivariate regression” multiple times, although this was not apparent. A true multivariate regression would have multiple covariates included in a single regression sharing a common independent variable(s). These covariates, collectively, have influence on the outcome based on their values and respective relationships with one another. What I saw given the tables, was a series of univariate regressions – this is not a multivariate regression, and not a model, and in that sense, is a bit misleading. Please add details to this to either strengthen if it is indeed a true multivariate regression model (including an equation of the proposed model), or correct the wording to make clear it’s in fact, univariate analyses.
- Ultimately, this seemed like a review or a general description of COVID-19 that provided more information that we already know. However, I believe some changes (albeit a bit more involved) will greatly strengthen the main message and provide very insightful information for the field. Specifically, to enhance what this paper has done, I believe key variables need to be added to maximize the results. These include:
  - Evaluating age-adjusted rates (including cases and deaths) if they are available; these values can then be used to more accurately compared across different geographic areas and increased the validity of the analysis.
  - The actual number of tests taken per person is critical here that is missing. Yes, the proportion of individuals who have been tested is there (I’m assuming these are per person, with at least one test). But a much more effective analysis would be to include the number of tests taken per person, if known, and in turn, will better reflect the insight regarding test positivity (the old saying is, the more you test, the more you find something…but the test rates will likely decline given the large denominator (tests taken).
  - With these updates, an actual model can be conducted and provide very meaningful insight into healthcare equity and access to care, especially if more effort is taken to demonstrate these values by ethnicity in space and time.

Minor Questions/Concerns:

- PLoS One is an open access journal and data sharing is encouraged (if not required). Please add more information and cite wherever a data source was used, and include a url is it has publicly available information.
- There are a few grammatical suggestions throughout, please review those accordingly.
- Please state why long term nursing home residents were not included in the analysis. And if possible, please provide more context to the individuals excluded from the study (per figure 1).
  - In general, figure captions 2 and 3 are too vague and need more information as well
- Quite a few papers regarding SES and COVID-19 are available that refer to many purported introductory comments and findings. Please add a few more where noted in track changes.
- Please address the majority of the minor questions/issues in the track changes

PLOS ONE

Comparison of socio-economic determinants of COVID-19 testing and positivity in Canada: a multi-provincial analysis.

--Manuscript Draft--

| Manuscript Number: | PONE-D-22-25812 |
| --- | --- |
| Article Type: | Research Article |
| Full Title: | Comparison of socio-economic determinants of COVID-19 testing and positivity in Canada: a multi-provincial analysis. |
| Short Title: | COVID-19 Testing and SDH |
| Corresponding Author: | Lilia Antonova, Ph.D.  University of Ottawa Faculty of Medicine Gatineau, QC CANADA |
| Keywords: | COVID-19 testing; COVID-19 positivity; SARS-CoV-2; social determinants of health; socioeconomic status; interprovincial analysis |
| Abstract: | Background : The effects of the COVID-19 pandemic have been more pronounced for socially disadvantaged populations. We sought to determine how access to SARS- CoV-2 testing and the likelihood of testing positive for COVID-19 were associated with demographic factors, socioeconomic status (SES) and social determinants of health (SDH) in three Canadian provinces.  Methods : An observational population-based cross-sectional study was conducted for the provinces of Ontario, Manitoba and New Brunswick between March 1, 2020 and April 27, 2021. After excluding residents of long-term care homes, those without current provincial health insurance and those who were tested for COVID-19 out of province, records from provincial healthcare administrative databases were reviewed for 16,900,661 healthcare users. We employed univariate and multivariate regression models to examine determinants of testing and test results.  Results : Female sex and urban residency were positively associated with testing, while female sex was negatively associated with test positivity. In New Brunswick and Ontario, individuals living in higher income areas were more likely to be tested, whereas in Manitoba higher income was negatively associated with both testing and positivity. High ethnocultural composition was associated with lower testing rates. Both high ethnocultural composition and high situational vulnerability increased the odds of testing positive for SARS-CoV-2.  Discussion : We observed that multiple demographic, income and SDH factors were associated with SARS-CoV-2 testing and test positivity. Barriers to healthcare access identified in this study specifically relate to COVID-19 testing but may reflect broader inequities for certain at-risk groups. |
| Order of Authors: | Lilia Antonova, Ph.D. |
|  | Chandy Somayaji, MSc |
|  | Jillian Cameron, MSc |
|  | Monica Sirski, PhD |
|  | Maria Sundaram, MSPH, PhD |
|  | James Ted McDonald, PhD |
|  | Sharmistha Mishra, MD, PhD |
|  | Jeffrey C Kwong, MD, MSc |
|  | Alan Katz |
|  | Stefan Baral |
|  | Lisa Caulley, MD, MPH |
|  | Andrew Calzavara, MSc |
|  | Martin Corsten, MD |

|  | Stephanie Johnson-Obaseki, MD, MSc |
| --- | --- |
| Additional Information: |  |
| Question | Response |
| Financial Disclosure  Enter a financial disclosure statement that describes the sources of funding for the work included in this submission. Review the [submission guidelines](http://journals.plos.org/plosone/s/submission-guidelines#loc-financial-disclosure-statement) for detailed requirements. View published research articles from [PLOS ONE](http://journals.plos.org/plosone/) for specific examples.  This statement is required for submission and will appear in the published article if the submission is accepted. Please make sure it is accurate. | Funding and Disclaimers  This work was supported by Public Health Ontario. This study was also supported by ICES, which is funded by an annual grant from the Ontario Ministry of Health (MOH) and the Ministry of Long-Term Care (MLTC). This study was supported by the Ontario Health Data Platform (OHDP), a Province of Ontario initiative to support Ontario’s ongoing response to COVID-19 and its related impacts. The analyses for Ontario and Manitoba were supported by the Canadian Institutes of Health Research (VR5- 172683). This work was supported by the Department of Health and Vitalité Health Network of the Province of New Brunswick under a contract with the New Brunswick Institute for Research, Data and Training at the University of New Brunswick. The results and conclusions are those of the authors and no official endorsement by the Government of New Brunswick or Vitalité Health Network was intended or should be inferred. The study sponsors did not participate in the design and conduct of the study; collection, management, analysis and interpretation of the data; preparation, review or approval of the manuscript; or the decision to submit the manuscript for publication.  Parts of this material are based on data and/or information compiled and provided by the Canadian Institute for Health Information (CIHI) and by Ontario Health (OH).  However, the analyses, conclusions, opinions and statements expressed herein are solely those of the authors, and do not reflect those of the funding or data sources; no endorsement by ICES, MOH, MLTC, OHDP, its partners, the Province of Ontario, CIHI or OH is intended or should be inferred. J.C.K. is supported by a Clinician-Scientist Award from the University of Toronto Department of Family and Community Medicine. SM is supported by a Tier 2 Canada Research Chair in Mathematical Modeling and Program Science. |
| Unfunded studies  Enter: The author(s) received no specific funding for this work.  Funded studies  Enter a statement with the following details:   - Initials of the authors who received each award - Grant numbers awarded to each author - The full name of each funder - URL of each funder website - Did the sponsors or funders play any role in the study design, data collection and analysis, decision to publish, or preparation of the manuscript? - NO - Include this sentence at the end of your statement: The funders had no role in study design, data collection and analysis, decision to publish, or preparation of the manuscript. - YES - Specify the role(s) played. |  |
| * typeset |  |
| Competing Interests  Use the instructions below to enter a competing interest statement for this submission. On behalf of all authors, disclose any [competing interests](http://journals.plos.org/plosone/s/competing-interests) that could be perceived to bias this work—acknowledging all financial support and any other relevant financial or non- financial competing interests. | The authors have declared that no completing interests exist. |

| This statement is required for submission and will appear in the published article if the submission is accepted. Please make sure it is accurate and that any funding sources listed in your Funding Information later in the submission form are also declared in your Financial Disclosure statement.  View published research articles from  [PLOS ONE](http://journals.plos.org/plosone/) for specific examples. |  |
| --- | --- |
| NO authors have competing interests  Enter: The authors have declared that no competing interests exist.  Authors with competing interests  Enter competing interest details beginning with this statement:  I have read the journal's policy and the authors of this manuscript have the following competing interests: [insert competing interests here] |  |
| * typeset |  |
| Ethics Statement  Enter an ethics statement for this submission. This statement is required if the study involved:   - Human participants - Human specimens or tissue - Vertebrate animals or cephalopods - Vertebrate embryos or tissues - Field research   Write "N/A" if the submission does not require an ethics statement.  General guidance is provided below. Consult the [submission guidelines](http://journals.plos.org/plosone/s/submission-guidelines#loc-guidelines-for-specific-study-types) for detailed instructions. Make sure that all | Ethics approval for this study was granted by the University of New Brunswick Research Ethics Board (New Brunswick), Ottawa Health Science Network Research Ethics Board (Ontario) and the University of Manitoba Health Research Ethics Board (Manitoba). The Manitoba ethics application was reviewed for data privacy and approved by the Government of Manitoba’s Health Information and Privacy Committee (File No. 2020/2021-32). |

| information entered here is included in the Methods section of the manuscript. |  |
| --- | --- |
| Format for specific study types  Human Subject Research (involving human participants and/or tissue)   - Give the name of the institutional review board or ethics committee that approved the study - Include the approval number and/or a statement indicating approval of this research - Indicate the form of consent obtained (written/oral) or the reason that consent was not obtained (e.g. the data were analyzed anonymously)   Animal Research (involving vertebrate animals, embryos or tissues)   - Provide the name of the Institutional Animal Care and Use Committee (IACUC) or other relevant ethics board that reviewed the study protocol, and indicate whether they approved this research or granted a formal waiver of ethical approval - Include an approval number if one was obtained - If the study involved non-human primates, add additional details about animal welfare and steps taken to ameliorate suffering - If anesthesia, euthanasia, or any kind of animal sacrifice is part of the study, include briefly which substances and/or methods were applied   Field Research  Include the following details if this study involves the collection of plant, animal, or other materials from a natural setting:   - Field permit number - Name of the institution or relevant body that granted permission |  |
|  |  |
| Data Availability  Authors are required to make all data underlying the findings described fully available, without restriction, and from the time of publication. PLOS allows rare exceptions to address legal and ethical | No - some restrictions will apply |

| concerns. See the [PLOS Data Policy](http://journals.plos.org/plosone/s/data-availability) and [FAQ](http://journals.plos.org/plosone/s/data-availability#loc-faqs-for-data-policy) for detailed information.  A Data Availability Statement describing where the data can be found is required at submission. Your answers to this question constitute the Data Availability Statement and will be published in the article, if accepted. |  |
| --- | --- |
| Important: Stating ‘data available on request from the author’ is not sufficient. If your data are only available upon request, select ‘No’ for the first question and explain your exceptional situation in the text box. |  |
| Do the authors confirm that all data underlying the findings described in their manuscript are fully available without restriction? |  |
| Describe where the data may be found in full sentences. If you are copying our sample text, replace any instances of XXX with the appropriate details.   - If the data are held or will be held in a public repository, include URLs, accession numbers or DOIs. If this information will only be available after acceptance, indicate this by ticking the box below. For example: All XXX files are available from the XXX database (accession number(s) XXX, XXX.). - If the data are all contained within the manuscript and/or Supporting Information files, enter the following: All relevant data are within the manuscript and its Supporting Information files. - If neither of these applies but you are able to provide details of access elsewhere, with or without limitations, please do so. For example:   Data cannot be shared publicly because of [XXX]. Data are available from the XXX Institutional Data Access / Ethics Committee (contact via XXX) for researchers who meet the criteria for access to confidential data. | Aggregate data underlying the results presented in this study are available from ICES [(https://www.ices.on.ca/).](http://www.ices.on.ca/)) ICES does not provide individual-level data. |

| The data underlying the results presented in the study are available from (include the name of the third party and contact information or URL).   - This text is appropriate if the data are owned by a third party and authors do not have permission to share the data.   * typeset |  |
| --- | --- |
| Additional data availability information: |  |

<https://www.editorialmanager.com/pone/download.aspx?id=31852626&guid=cbb59d52-c1f5-4e0a-acc4-4d1e2f487ef1&scheme=1>Manuscript	[Click here to access/download;Manuscript;Manuscript September 16 2022.docx](https://www.editorialmanager.com/pone/download.aspx?id=31852626&guid=cbb59d52-c1f5-4e0a-acc4-4d1e2f487ef1&scheme=1)

1 **Comparison of socio-economic determinants of COVID-19 testing and positivity in Canada: a**
2 **multi-provincial analysis.**
3 **Authors:**
4 Lilia Antonova PhD^a,b^, Chandy Somayaji MSc^c^, Jillian Cameron MSc^c^, Monica Sirski PhD^d^, Maria
5 Sundaram MSPH PhD^e,f^, James Ted McDonald PhD^g^, Sharmistha Mishra MD PhD^h,i^, Jeffrey C. Kwong
6 MD MSc^f,j^, Alan Katz^d^, Stefan Baral^k^, Lisa Caulley MD MPH FRCSC^a,b,l^, Andrew Calzavara MSc^f^,
7 *Martin Corsten FRCSC^m^ and *Stephanie Johnson-Obaseki MSc, FRCSC^a,b^
8 * indicates co-senior authorship
9 a Department of Otolaryngology – Head and Neck Surgery, University of Ottawa, ON, Canada
10 b Clinical Epidemiology Program, Ottawa Hospital Research Institute, ON, Canada
11 c New Brunswick Institute for Research, Data, and Training, University of New Brunswick, NB, Canada
12 d Manitoba Centre for Health Policy, University of Manitoba, MB, Canada
13 e Center for Clinical Epidemiology and Population Health, Marshfield Clinic Research Institute, WI,
14 USA
15 f ICES, Toronto, ON, Canada
16 g Department of Political Science, University of New Brunswick, NB, Canada
17 h Department of Medicine, University of Toronto, ON, Canada
18 i MAP Center for Urban Health Solutions, St. Michael’s Hospital, Unity Health Toronto, ON, Canada
19 j Dalla Lana School of Public Health, University of Toronto, ON, Canada
20 k Department of Epidemiology, Johns Hopkins University School of Public Health, MD, USA
21 l Department of Epidemiology, Erasmus University Medical Center Rotterdam, Netherlands
22 m Division of Otolaryngology – Head and Neck Surgery, Dalhousie University, NS, Canada

23

24 **Corresponding Author:**
25 Dr. Stephanie Johnson-Obaseki
26 S3 501 Smyth Rd
27 Ottawa ON
28 613 798 5555 ext 78596
29 Email: [stjohnson@toh.ca](mailto:stjohnson@toh.ca)

30

31 **Funding and Disclaimers**
32 This work was supported by Public Health Ontario. This study was also supported by ICES, which is funded by an
33 annual grant from the Ontario Ministry of Health (MOH) and the Ministry of Long-Term Care (MLTC). This study
34 was supported by the Ontario Health Data Platform (OHDP), a Province of Ontario initiative to support Ontario’s
35 ongoing response to COVID-19 and its related impacts. The analyses for Ontario and Manitoba were supported by
36 the Canadian Institutes of Health Research (VR5-172683). This work was supported by the Department of Health
37 and Vitalité Health Network of the Province of New Brunswick under a contract with the New Brunswick Institute
38 for Research, Data and Training at the University of New Brunswick. The results and conclusions are those of the
39 authors and no official endorsement by the Government of New Brunswick or Vitalité Health Network was intended
40 or should be inferred. The study sponsors did not participate in the design and conduct of the study; collection,
41 management, analysis and interpretation of the data; preparation, review or approval of the manuscript; or the
42 decision to submit the manuscript for publication. Parts of this material are based on data and/or information
43 compiled and provided by the Canadian Institute for Health Information (CIHI) and by Ontario Health (OH).
44 However, the analyses, conclusions, opinions and statements expressed herein are solely those of the authors, and do
45 not reflect those of the funding or data sources; no endorsement by ICES, MOH, MLTC, OHDP, its partners, the
46 Province of Ontario, CIHI or OH is intended or should be inferred. J.C.K. is supported by a Clinician-Scientist
47 Award from the University of Toronto Department of Family and Community Medicine. SM is supported by a Tier
48 2 Canada Research Chair in Mathematical Modeling and Program Science.

# 49 Socio-economic determinants of COVID-19 testing and positivity in Canada: a multi-

50 **province analysis.**

51

52

53	**ABSTRACT: (242 words)**

54

55 ***Background***: The effects of the COVID-19 pandemic have been more pronounced for socially
56 disadvantaged populations. We sought to determine how access to SARS-CoV-2 testing and the
57 likelihood of testing positive for COVID-19 were associated with demographic factors,
58 socioeconomic status (SES) and social determinants of health (SDH) in three Canadian
59 provinces.
60 ***Methods***: An observational population-based cross-sectional study was conducted for the
61 provinces of Ontario, Manitoba and New Brunswick between March 1, 2020 and April 27, 2021.
62 After excluding residents of long-term care homes, those without current provincial health
63 insurance and those who were tested for COVID-19 out of province, records from provincial
64 healthcare administrative databases were reviewed for 16,900,661 healthcare users. We
65 employed univariate and multivariate regression models to examine determinants of testing and
66 test results.
67 ***Results***: Female sex and urban residency were positively associated with testing, while female
68 sex was negatively associated with test positivity. In New Brunswick and Ontario, individuals
69 living in higher income areas were more likely to be tested, whereas in Manitoba higher income
70 was negatively associated with both testing and positivity. High ethnocultural composition was
71 associated with lower testing rates. Both high ethnocultural composition and high situational
72 vulnerability increased the odds of testing positive for SARS-CoV-2.
73 ***Discussion***: We observed that multiple demographic, income and SDH factors were associated
74 with SARS-CoV-2 testing and test positivity. Barriers to healthcare access identified in this
75 study specifically relate to COVID-19 testing but may reflect broader inequities for certain at-
76 risk groups.

77

78

79

80

81

82

83

84

85

86

87

88

89

90

91

92

93

# 94 Introduction

95 The COVID-19 pandemic has highlighted the disproportionate vulnerability of certain
96 populations to unanticipated health disasters. Even in high-income countries such as the United
97 Kingdom and the United States increased positivity rates and poorer health outcomes, including
98 death, from COVID-19 infection have been identified in populations with lower SES.^1-4^
99 Previous work has identified some of the social determinants of health (SDH) contributing to
100 socioeconomic differences in SARS-CoV-2 testing and infection rates in the United States,
101 including household density, income, race, essential work, and health insurance status.^5-8^ As
102 health coverage was found to be an important factor associated with COVID-19 positivity in the
103 United States, it is likely that the relative contribution of SDH and SES would be different in a
104 country such as Canada, where there is free access to universal healthcare coverage.
105 Additionally, as public health measures and health care delivery are largely provincial
106 responsibilities in Canada, the relative effects of SDH and SES may vary by province.
107 An important determinant of COVID-19 outcomes is access to SARS-CoV-2 testing.^9-10^ Equity
108 in testing access contributes to timely disease management, helping to improve disease outcomes
109 and to lighten the burden on the healthcare system.^9,11-12^ An optimal testing strategy would make
110 SARS-CoV-2 testing more available for socially disadvantaged groups to address the higher
111 rates of infection in these populations. SES plays a role in SARS-CoV-2 testing disparities, with
112 the most SARS-CoV-2-positive communities exhibiting some of the lowest testing rates in the
113 United States.^13-14^ A similar relationship between high-risk communities and lower testing rates
114 was demonstrated in Ontario, Canada.^15^ The generalizability of this finding across various
115 Canadian provinces remains a research question of interest.
116 To address these gaps in knowledge, we first sought to examine differences across provinces
117 (New Brunswick, Manitoba, and Ontario) in testing rates and test positivity. Second, we sought
118 to examine if demographic, medical history, SES and SDH variables were associated with testing
119 rates and test positivity across these three provinces using available census and health
120 administrative data.

121

# 122 Materials and Methods

123 *Study design and study population*
124 We conducted a population-based, cross-sectional, observational study across three Canadian
125 provinces differing in their sizes and population distributions as well as in the relative impact of
126 COVID-19 - New Brunswick (NB) , Manitoba (MB), and Ontario (ON). We used laboratory and
127 health administrative databases in each province as well as area-level SES and SDH
128 characteristics derived from census data. Data were de-identified prior to linkage. Ethics
129 approval for this study was granted by the University of New Brunswick Research Ethics Board
130 (New Brunswick), Ottawa Health Science Network Research Ethics Board (Ontario) and the
131 University of Manitoba Health Research Ethics Board (Manitoba). The Manitoba ethics
132 application was reviewed for data privacy and approved by the Government of Manitoba’s
133 Health Information and Privacy Committee (File No. 2020/2021-32).
134 The cohort in each province included all individuals registered with their respective provincial
135 health system living as of March 1, 2020, as well as those born between March 1, 2020 and
136 October 1, 2020. The study period was from March 1, 2020 to April 27, 2021. Long-term care
137 residents, current residents without a valid healthcare card for the period of interest and out-of-
138 province residents who accessed SARS-CoV-2 testing were excluded.
139 *Data*
140 Testing and PCR test result information was identified from provincial databases (Supplemental
141 Table 1). Manitoba data were accessed from the Manitoba Population Research Repository
142 housed at the Manitoba Centre for Health Policy, New Brunswick data were accessed from the
143 New Brunswick Institute for Research, Data, and Training (NB-IRDT), and Ontario data were
144 linked using unique encoded identifiers and analyzed at ICES (formerly the Institute for Clinical
145 Evaluative Sciences). We evaluated test positivity as follows: first we considered whether an
146 individual was tested; then, of the tested individuals, we considered if their test result was
147 positive or negative. If an individual had more than one test in the time period of interest, we
148 selected the first positive test or, if all tests were negative, the first negative test.
149 To explore factors contributing to testing and test positivity, we examined the role of relevant
150 demographic, medical history, socioeconomic and SDH variables determined from provincial
151 health administrative data (Supplemental Table 1). *Demographic variables* examined included
152 age and sex. Information about these variables was obtained from citizen databases containing
153 healthcare information. *Medical history variables* included the number of hospital admissions in
154 the 3 years prior to the time period of interest (reflecting underlying health status) and
155 comorbidities (including cancer [NB and ON], asthma [MB and ON], dementia and frailty [MB
156 and ON], heart disease [MB and ON], as well as chronic obstructive pulmonary disease,
157 hypertension, and diabetes [all provinces]). Hospital admissions (0, 1, 2 or ≥3) and comorbidities
158 were defined using hospital discharge abstracts and physician billing claims. *Variables*
159 *indicative of SES* included rural or urban residence and area-level quintiles for income (after tax
160 income per person equivalent). *Variables indicative of SDH* included four measures from the
161 Canadian Index of Multiple Deprivation (CIMD). Area-level variables were obtained by linking
162 postal code to dissemination areas using the Postal Code Conversion File (version 7B for NB and
163 ON, and 7D for MB). The CIMD is a neighborhood-level index based on census data introduced
164 by Statistics Canada in 2016.^16^ There are four SDH variables evaluated in the CIMD: residential
165 instability (for example, the proportion of individuals who have moved in the past 5 years and
166 the number of apartment buildings in the neighborhood); economic dependency (for example,
167 the proportion of the population who are employed and the proportion receiving government
168 assistance); ethnocultural composition (for example, the proportion of the population who
169 identify as visible minorities or are recent immigrants); and situational vulnerability (for
170 example, the proportion of population who are Aboriginal or who have not attained a high school
171 diploma). All four components of the CIMD are ranked in levels from 1 to 5, with higher ranking
172 indicating a greater presence of that component. We used the national level values for all the
173 indices to allow for comparison between provinces.
174 *Analysis*
175 Each province undertook their own data analyses using standard variable definitions and protocol
176 training. We examined two separate outcomes: testing and test positivity and therefore we
177 conducted parallel analyses for comparing a) tested versus untested individuals and b) tested
178 individuals who tested positive versus those who tested negative. All analyses were conducted at
179 the individual level, examining the effects of the demographic and neighborhood variables on
180 each outcome variable for each province. Descriptive statistics were calculated for all
181 demographic variables, SES and SDH. Statistical differences between demographic variables
182 were evaluated using Welch’s t-test for continuous variables and Fisher’s chi-squared test for
183 categorical variables. To examine unadjusted determinants of testing and test result, we
184 conducted univariate logistic regression models on each variable. We also analyzed all of the
185 variables in a fully adjusted multivariate model. Statistical analyses were conducted using SAS

186

187

v. 9.4 (SAS 2013 Statistical Analysis Software, SAS Institute Inc, Cary, NC).
   188 **Results**
   189 Data from provincial surveillance databases indicated that many individuals enrolled in the
   190 provincial health insurance systems received testing over the study period: 25% for NB
   191 (N=159,150), 40% for MB (N=401,251) and 49% for ON (N=4,792,449) (Figure 1). Of those
   192 tested, 1% (N=1645), 9% (N=35,458) and 5% (N=258,769), tested positive for SARS-CoV-2
   193 (NB, MB and ON, respectively) (Figure 1). Comparison of the tested and untested populations
   194 demonstrated that they differed in the distribution of age, sex and place of residence (Table 1)
   195 (p<0.001 for all variables for all provinces). The same was found for those who tested positive,
   196 as compared to those who tested negative (Table 1) (p<0.0001 for all variables for all provinces).
   197 **Figure 1:** Flow diagram for untested and COVID-19 tested groups, and negative or positive test
   198 results for the provinces of New Brunswick (NB), Manitoba (MB), and Ontario (ON). 199

|  | **New Brunswick** | | | | **Manitoba** | | | | **Ontario** | | | |
| --- | --- | --- | --- | --- | --- | --- | --- | --- | --- | --- | --- | --- |
|  | **All individuals** | | **Tested individuals** | | **All individuals** | | **Tested individuals** | | **All individuals** | | **Tested individuals** | |
|  | **Untested control** | **All tested** | **Tested negative** | **Tested positive** | **Untested control** | **All tested** | **Tested negative** | **Tested positive** | **Untested control** | **All tested** | **Tested negative** | **Tested positive** |
| **N** | 635 677 | 159 150 | 157 505 | 1645 | 1 009 004 | 401 251 | 365 793 | 35 458 | 9 903 130 | 4 792 449 | 4 533 688 | 258 761 |
| **Age: mean** | 44.81 | 41.11 | 41.05 | 46.00 | 39.27 | 38.89 | 38.90 | 38.54 | 41.09 | 40.16 | 40.21 | 39.19 |
| **(SD)** | (23.61) | (22.85) | (22.84) | (22.84) | (23.87) | (22.47) | (22.44) | (22.81) | (23.25) | (22.13) | (22.24) | (20.22) |
| **Ages: 0-4 (%)** | 4.02 | 5.21 | 5.24 | 2.81 | 6.56 | 4.94 | 5.00 | 4.35 | 5.27 | 4.53 | 4.62 | 3.00 |
| **Ages: 5-19** | 14.74 | 15.22 | 15.26 | 11.53 | 19.33 | 16.65 | 16.49 | 18.29 | 17.25 | 15.06 | 15.02 | 15.86 |
| **(%)** |  |  |  |  |  |  |  |  |  |  |  |  |
| **Ages: 20-34** | 16.49 | 20.11 | 20.12 | 18.66 | 19.12 | 24.41 | 24.31 | 25.51 | 18.56 | 23.69 | 23.62 | 24.92 |
| **(%)** |  |  |  |  |  |  |  |  |  |  |  |  |
| **Ages: 35-49** | 18.02 | 21.32 | 21.33 | 20.55 | 18.48 | 21.84 | 21.98 | 20.35 | 19.41 | 21.21 | 21.10 | 23.22 |
| **(%)** |  |  |  |  |  |  |  |  |  |  |  |  |
| **Ages: 50-64** | 22.92 | 20.84 | 20.79 | 25.81 | 18.92 | 17.59 | 17.61 | 17.36 | 21.06 | 20.43 | 20.34 | 22.08 |
| **(%)** |  |  |  |  |  |  |  |  |  |  |  |  |
| **Ages: 65-74** | 13.98 | 9.97 | 9.97 | 9.64 | 10.27 | 7.59 | 7.72 | 6.24 | 10.91 | 8.34 | 8.44 | 6.63 |
| **(%)** |  |  |  |  |  |  |  |  |  |  |  |  |
| **Ages: 75-84** | 6.99 | 5.00 | 5.00 | 5.78 | 5.08 | 4.21 | 4.23 | 3.93 | 5.63 | 4.39 | 4.47 | 2.99 |
| **(%)** |  |  |  |  |  |  |  |  |  |  |  |  |
| **Ages: 85+ (%)** | 2.84 | 2.32 | 2.29 | 5.37 | 2.22 | 2.78 | 2.66 | 3.97 | 1.91 | 2.35 | 2.41 | 1.31 |
| **Males (%)** | 50.69 | 44.17 | 44.14 | 46.67 | 51.20 | 46.08 | 45.79 | 49.05 | 50.63 | 46.28 | 45.96 | 51.91 |
| **Females (%)** | 49.31 | 55.83 | 55.86 | 53.33 | 48.80 | 53.92 | 54.21 | 50.95 | 49.37 | 53.72 | 54.05 | 48.09 |
| **Rural residence (%)** | 38.15 | 31.11 | 31.14 | 28.21 | 28.76 | 28.15 | 27.66 | 33.13 | 10.27 | 9.52 | 9.86 | 3.64 |
| **Urban residence (%)** | 61.85 | 68.89 | 68.86 | 71.79 | 71.24 | 71.85 | 72.34 | 66.87 | 89.73 | 90.48 | 90.14 | 96.36 |
| **Household income quintile (%)** | | | | | | | | | | | | |

| **(lowest** | 20.30 | 19.25 | 19.22 | 21.70 | 20.40 | 23.17 | 21.72 | 38.10 | 19.54 | 18.95 | 18.60 | 25.11 |
| --- | --- | --- | --- | --- | --- | --- | --- | --- | --- | --- | --- | --- |
| **income) 1** |  |  |  |  |  |  |  |  |  |  |  |  |
| **2** | 19.83 | 18.80 | 18.79 | 20.30 | 19.00 | 18.55 | 18.56 | 18.39 | 19.59 | 19.05 | 18.90 | 21.57 |
| **3** | 20.09 | 19.36 | 19.35 | 20.36 | 19.76 | 18.65 | 18.95 | 15.54 | 20.06 | 19.93 | 19.84 | 21.48 |
| **4** | 19.60 | 20.03 | 20.06 | 17.63 | 21.41 | 19.98 | 20.42 | 15.47 | 20.02 | 20.27 | 20.45 | 17.24 |
| **(highest** | 19.81 | 22.15 | 22.18 | 19.57 | 19.46 | 19.66 | 20.35 | 12.50 | 19.73 | 20.93 | 21.36 | 13.45 |
| **income) 5** |  |  |  |  |  |  |  |  |  |  |  |  |
| **Underlying** |  |  |  |  |  |  |  |  |  |  |  |  |
| **chronic** |  |  |  |  |  |  |  |  |  |  |  |  |
| **health** |  |  |  |  |  |  |  |  |  |  |  |  |
| **conditions** |  |  |  |  |  |  |  |  |  |  |  |  |
| **(%)** |  |  |  |  |  |  |  |  |  |  |  |  |
| **COPD** | 7.14 | 7.69 | 7.68 | 8.09 | 0.36 | 0.81 | 0.79 | 0.95 | 1.74 | 2.35 | 2.42 | 1.18 |
| **Hypertension** | 25.69 | 22.67 | 22.61 | 28.09 | 15.94 | 16.42 | 16.29 | 17.68 | 21.06 | 20.55 | 20.58 | 20.08 |
| **Diabetes** | 10.51 | 10.04 | 10.01 | 12.77 | 6.88 | 8.73 | 8.44 | 11.74 | 9.99 | 10.17 | 10.04 | 12.52 |
| **Hospital** |  |  |  |  |  |  |  |  |  |  |  |  |
| **admissions,** | 1.86 (1.45) | 2.03 (1.83) | 2.03 (1.83) | 2.17 (1.85) | 0.19 (0.61) | 0.34 (0.98) | 0.33 (0.97) | 0.39 (1.13) | 0.17 (0.54) | 0.26 (0.85) | 0.27 (0.87) | 0.17 (0.60) |
| **past 3 years:** |  |  |  |  |  |  |  |  |  |  |  |  |
| **Mean(SD)** |  |  |  |  |  |  |  |  |  |  |  |  |
| **CIMD –** |  |  |  |  |  |  |  |  |  |  |  |  |
| **Residential** | N=631 440 | N=158 015 | N =156 380 | N =1635 | N=995 798 | N=395 783 | N=360 782 | N=35 001 | N=9 791 430 | N=4 747 784 | N=4 483 422 | N=255 665 |
| **instability** |  |  |  |  |  |  |  |  |  |  |  |  |
| **quintiles (%)** |  |  |  |  |  |  |  |  |  |  |  |  |
| **1 (least** | 21.40 | 21.82 | 21.84 | 20.06 | 21.73 | 23.02 | 22.02 | 33.36 | 20.89 | 20.56 | 20.71 | 17.86 |
| **deprived)** |  |  |  |  |  |  |  |  |  |  |  |  |
| **2** | 25.64 | 24.29 | 24.31 | 22.32 | 17.78 | 17.30 | 17.62 | 13.99 | 23.12 | 22.61 | 22.62 | 22.45 |
| **3** | 22.68 | 22.71 | 22.70 | 23.30 | 15.70 | 15.89 | 16.16 | 13.17 | 19.25 | 19.46 | 19.44 | 19.80 |
| **4** | 16.60 | 17.63 | 17.61 | 19.33 | 20.26 | 18.67 | 19.03 | 14.95 | 16.71 | 17.39 | 17.46 | 16.11 |
| **5 (most** | 13.68 | 13.55 | 13.54 | 14.98 | 24.53 | 25.12 | 25.17 | 24.55 | 20.03 | 19.99 | 19.77 | 23.78 |
| **deprived)** |  |  |  |  |  |  |  |  |  |  |  |  |

| **CIMD –** |  |  |  |  |  |  |  |  |  |  |  |  |
| --- | --- | --- | --- | --- | --- | --- | --- | --- | --- | --- | --- | --- |
| **Economic** |  |  |  |  |  |  |  |  |  |  |  |  |
| **dependency** |  |  |  |  |  |  |  |  |  |  |  |  |
| **quintiles (%)** |  |  |  |  |  |  |  |  |  |  |  |  |
| **1 (least** | 11.96 | 13.42 | 13.49 | 6.61 | 18.51 | 18.92 | 18.73 | 20.88 | 22.46 | 23.11 | 23.16 | 22.15 |
| **deprived)** |  |  |  |  |  |  |  |  |  |  |  |  |
| **2** | 14.15 | 14.84 | 14.87 | 11.74 | 20.24 | 20.67 | 20.69 | 20.44 | 22.73 | 22.97 | 22.81 | 25.86 |
| **3** | 19.25 | 19.22 | 19.22 | 19.39 | 19.02 | 19.82 | 19.67 | 21.29 | 21.21 | 21.13 | 21.09 | 21.80 |
| **4** | 23.28 | 23.08 | 23.00 | 30.70 | 19.29 | 20.12 | 19.96 | 21.83 | 18.66 | 18.28 | 18.24 | 19.02 |
| **5 (most** | 31.35 | 29.44 | 29.42 | 31.56 | 22.93 | 20.47 | 20.95 | 15.55 | 14.94 | 14.51 | 14.70 | 11.17 |
| **deprived)** |  |  |  |  |  |  |  |  |  |  |  |  |
| **CIMD –** |  |  |  |  |  |  |  |  |  |  |  |  |
| **Ethnocultural** |  |  |  |  |  |  |  |  |  |  |  |  |
| **composition** |  |  |  |  |  |  |  |  |  |  |  |  |
| **(%)** |  |  |  |  |  |  |  |  |  |  |  |  |
| **1 (least** | 44.07 | 44.33 | 44.28 | 48.69 | 13.61 | 13.02 | 13.26 | 10.53 | 7.34 | 7.28 | 7.55 | 2.54 |
| **deprived)** |  |  |  |  |  |  |  |  |  |  |  |  |
| **2** | 36.10 | 35.22 | 35.27 | 30.76 | 19.62 | 20.57 | 20.74 | 18.91 | 15.41 | 15.48 | 16.00 | 6.36 |
| **3** | 12.72 | 13.33 | 13.33 | 13.27 | 18.97 | 20.93 | 20.97 | 20.49 | 18.80 | 19.81 | 20.30 | 11.07 |
| **4** | 4.94 | 5.21 | 5.21 | 5.14 | 20.86 | 20.83 | 20.93 | 19.88 | 22.22 | 23.32 | 23.54 | 19.43 |
| **5 (most** | 2.16 | 1.91 | 1.91 | 2.14 | 26.93 | 24.65 | 24.11 | 30.20 | 36.23 | 34.11 | 32.61 | 60.60 |
| **deprived)** |  |  |  |  |  |  |  |  |  |  |  |  |
| **CIMD –** |  |  |  |  |  |  |  |  |  |  |  |  |
| **Situational** |  |  |  |  |  |  |  |  |  |  |  |  |
| **vulnerability** |  |  |  |  |  |  |  |  |  |  |  |  |
| **(%)** |  |  |  |  |  |  |  |  |  |  |  |  |
| **1 (least** | 16.32 | 18.97 | 19.02 | 14.25 | 13.93 | 14.00 | 14.47 | 9.15 | 30.27 | 30.74 | 31.04 | 25.38 |
| **deprived)** |  |  |  |  |  |  |  |  |  |  |  |  |
| **2** | 16.76 | 16.96 | 17.02 | 11.68 | 20.02 | 19.14 | 19.67 | 13.59 | 22.3 | 22.49 | 22.49 | 22.51 |
| **3** | 14.97 | 15.68 | 15.67 | 17.00 | 16.68 | 15.23 | 15.44 | 13.10 | 18.03 | 17.98 | 17.93 | 18.74 |
| **4** | 24.10 | 23.55 | 23.46 | 32.11 | 20.32 | 19.05 | 19.24 | 17.11 | 15.39 | 15.15 | 15.08 | 16.39 |
| **5 (most** | 27.84 | 24.84 | 24.84 | 24.95 | 29.06 | 32.57 | 31.17 | 47.05 | 14.01 | 13.64 | 13.45 | 16.97 |
| **deprived)** |  |  |  |  |  |  |  |  |  |  |  |  |

200

201 In all provinces, the 20-34 and 35-49 age groups were most likely to be tested (Table 2, Figure
202 2). Children aged 5-19 were most likely to test positive in Manitoba and Ontario, whereas the
203 85+ age group was associated with the highest test positivity in New Brunswick (Table 3, Figure
204 3). In all provinces, females were significantly more likely to be tested than males and less likely
205 to test positive (Tables 2-3, Figures 2-3). Finally, urban residents were significantly more likely
206 to be tested in all provinces, and more likely to test positive in New Brunswick and Manitoba
207 (Tables 2-3, Figures 2-3).
208 **Figure 2:** Odds ratios for being tested, multivariate models.
209 **Figure 3:** Odds ratios for testing positive, multivariate models.

210

211 **Table 2:** Odds ratios and 95% confidence intervals for multivariable regression models of the
212 odds of SARS-CoV-2 testing in New Brunswick (NB), Manitoba (MB), and Ontario (ON).
213 Bold=significant.

| **Variable** | **NB** | **MB** | **ON** |
| --- | --- | --- | --- |
|  | **0.78 (0.75,** | **1.40 (1.37,** | **1.03 (1.03,** |
| Age group: 5-19 vs 0-4 | **0.80)** | **1.43)** | **1.04)** |
|  | **0.89 (0.86,** | **1.98 (1.95,** | **1.47 (1.46,** |
| Age group: 20-34 vs 0-4 | **0.91)** | **2.02)** | **1.47)** |
|  | **0.83 (s0.81,** | **1.83 (1.79,** | **1.24 (1.23,** |
| Age group: 35-49 vs 0-4 | **0.86)** | **1.86)** | **1.24)** |
|  | **0.61 (0.59,** | **1.36 (1.33,** | **1.05 (1.05,** |
| Age group: 50-64 vs 0-4 | **0.62)** | **1.38)** | **1.05)** |
|  | **0.42 (0.41,** | **0.97 (0.95,** | **0.76 (0.75,** |
| Age group: 65-74 vs 0-4 | **0.44)** | **0.99)** | **0.76)** |
|  | **0.38 (0.36,** | **0.92 (0.90,** | **0.71 (0.71,** |
| Age group: 75-84 vs 0-4 | **0.39)** | **0.94)** | **0.72)** |
|  | **0.40 (0.39,** | 0.99 (0.95, | **1.04 (1.04,** |
| Age group: 85+ vs 0-4 | **0.42)** | 1.02) | **1.05)** |
| Sex: Female vs Male | **1.28 (1.27,**  **1.30)** | **1.20 (1.20,**  **1.21)** | **1.09 (1.09,**  **1.09)** |
|  | 1.02 (1.00, | **0.90 (0.89,** | **0.94 (0.94,** |
| Income quintile: 2 vs 1 | 1.04) | **0.91)** | **0.94)** |
|  | **1.08 (1.06,** | **0.87 (0.86,** | **1.00 (0.99,** |
| Income quintile: 3 vs 1 | **1.11)** | **0.89)** | **1.00)** |

| Income quintile: 4 vs 1  Income quintile: 5 vs 1 | **1.20 (1.17,**  **1.23)**  **1.38 (1.34,**  **1.42)** | **0.87 (0.86,**  **0.89)**  **0.87 (0.85,**  **0.88)** | **1.05 (1.04,**  **1.05)**  **1.12 (1.11,**  **1.12)** |
| --- | --- | --- | --- |
| Rurality: Urban vs Rural | **1.45 (1.43,**  **1.47)** | **1.12 (1.11,**  **1.14)** | **1.05 (1.05,**  **1.05)** |
|  | **1.37 (1.35,** | **1.41 (1.39,** | **0.84 (0.83,** |
| Hospital admissions: 1 vs 0 | **1.39)** | **1.42)** | **0.84)** |
|  | **1.52 (1.49,** | **1.67 (1.63,** | **1.06 (1.05,** |
| Hospital admissions: 2 vs 0 | **1.56)** | **1.70)** | **1.07)** |
| Hospital admissions: ≥3 vs 0 | **1.87 (1.82,** | **2.44 (2.37,** | **1.74 (1.72,** |
|  | **1.92)** | **2.51)** | **1.75)** |
|  | **1.34 (1.31,** | **1.60 (1.51,** | **1.38 (1.37,** |
| Comorbidities: COPD | **1.37)** | **1.69)** | **1.39)** |
|  | **1.08 (1.06,** | **1.12 (1.11,** | **1.10 (1.10,** |
| Comorbidities: hypertension | **1.09)** | **1.14)** | **1.11)** |
|  | **1.11 (1.09,** | **1.30 (1.28,** | **1.10 (1.09,** |
| Comorbidities: diabetes | **1.13)** | **1.32)** | **1.10)** |
| CIMD Residential instability: 2 vs 1 | **1.04 (1.03,** | **0.93 (0.92,** | **0.95 (0.95,** |
|  | **1.06)** | **0.94)** | **0.96)** |
| CIMD Residential instability: 3 vs 1 | **1.15 (1.13,** | **0.97 (0.96,** | **1.01 (1.01,** |
|  | **1.17)** | **0.99)** | **1.01)** |
| CIMD Residential instability: 4 vs 1 | **1.23 (1.20,** | **0.91 (0.89,** | **1.07 (1.06,** |
|  | **1.25)** | **0.92)** | **1.07)** |
| CIMD Residential instability: 5 vs 1 | **1.11 (1.08,** | **0.94 (0.93,** | **1.04 (1.04,** |
|  | **1.14)** | **0.95)** | **1.04)** |
|  | 1.01 (0.99, | **0.97 (0.95,** | **1.01 (1.01,** |
| CIMD Economic dependency: 2 vs 1 | 1.03) | **0.98)** | **1.01)** |
|  | 1.02 (0.99, | **1.01 (1.00,** | 1.00 (1.00, |
| CIMD Economic dependency: 3 vs 1 | 1.04) | **1.03)** | 1.00) |
|  | **1.08 (1.05,** | 0.99 (0.98, | **0.99 (0.99,** |
| CIMD Economic dependency: 4 vs 1 | **1.10)** | 1.01) | **1.00)** |
|  | **1.11 (1.09,** | **0.87 (0.86,** | **1.00 (1.00,** |
| CIMD Economic dependency: 5 vs 1 | **1.14)** | **0.88)** | **1.00)** |
| CIMD Ethnocultural composition: 2 vs 1 | **0.95 (0.94,** | 1.01 (0.99, | **1.02 (1.02,** |
|  | **0.97)** | 1.02) | **1.02)** |
| CIMD Ethnocultural composition: 3 vs 1 | **0.93 (0.92,** | **1.03 (1.02,** | **1.03 (1.03,** |
|  | **0.96)** | **1.05)** | **1.03)** |
| CIMD Ethnocultural composition: 4 vs 1 | **0.90 (0.88,** | **0.90 (0.88,** | **1.01 (1.00,** |
|  | **0.93)** | **0.91)** | **1.01)** |
| CIMD Ethnocultural composition: 5 vs 1 | **0.77 (0.74,** | **0.76 (0.75,** | **0.92 (0.92,** |
|  | **0.81)** | **0.77)** | **0.92)** |
| CIMD Situational vulnerability: 2 vs 1 | **0.94 (0.93,** | **0.97 (0.96,** | **1.00 (1.00,** |
|  | **0.96)** | **0.99)** | **1.01)** |
| CIMD Situational vulnerability: 3 vs 1 | **1.05 (1.03,** | **0.92 (0.91,** | **1.01 (1.01,** |
|  | **1.08)** | **0.94)** | **1.01)** |
| CIMD Situational vulnerability: 4 vs 1 | **1.03 (1.01,** | **0.92 (0.90,** | **1.01 (1.00,** |
|  | **1.05)** | **0.93)** | **1.01)** |

CIMD Situational vulnerability: 5 vs 1	1.02 (1.00,	**1.03 (1.01,	1.00 (0.99,**

1.05)	**1.05)	1.00)**

214 Adjusted odds ratios and 95% CIs are presented. The adjusted model contains all variables
215 listed in the table and does not contain additional covariates that are not listed in the table.

216

217 **Table 3:** Odds ratios and confidence intervals for multivariable regression models of the odds of
218 testing positive for SARS-CoV-2 (among tested individuals), in New Brunswick (NB), Manitoba
219 (MB), and Ontario (ON).

**Variable	NB	MB	ON**

Age group: 5-19 vs _0-4_	**1.42 (1.02,	1.34 (1.26,	1.53 (1.49,**

**1.96)	1.42)	1.57)**

Age group: 20-34 vs _0-4_	**1.71 (1.25,	1.29 (1.22,	1.44 (1.40,**

**2.34)	1.37)	1.48)**

Age group: 35-49 vs _0-4_	**1.81 (1.33,	1.17 (1.10,	1.52 (1.48,**

**2.47)	1.25)	1.56)**

Age group: 50-64 vs _0-4_	**2.22 (1.63,	1.21 (1.13,	1.52 (1.48,**

**3.03)	1.29)	1.56)**

Age group: 65-74 vs 0-4	**1.75 (1.24,**	0.95 (0.88, 1.02)	**1.19 (1.15,**

**2.47)	1.22)**

Age group: 75-84 vs 0-4	**2.16 (1.48,**	0.93 (0.85, 1.02)	**1.05 (1.01,**

**3.14)	1.09)**

Age group: 85+ vs _0-4_	**4.42 (3.00,	1.15 (1.03,	0.90 (0.86,**

**6.50)	1.29)	0.94)**

Sex: Female vs Male	**0.90 (0.81,	0.87 (0.85,	0.81 (0.80,**

**0.99)	0.89)	0.81)**

Income quintile: 2 vs _1_	**0.68 (0.65,	0.84 (0.83,**

0.96 (0.81, 1.14)	**0.70)	0.86)**

Income quintile: 3 vs _1_	**0.59 (0.56,	0.77 (0.75,**

1.10 (0.91, 1.33)	**0.62)	0.78)**

Income quintile: 4 vs _1_	**0.55 (0.52,	0.63 (0.62,**

1.04 (0.84, 1.28)	**0.57)	0.65)**

Income quintile: 5 vs _1_	**0.45 (0.43,	0.55 (0.54,**

1.24 (0.99, 1.65)	**0.48)	0.57)**

Rurality: Urban vs Rural	**1.46 (1.29,	0.76 (0.73,	1.31 (1.28,**

**1.65)	0.79)	1.35)**

Hospital admissions: 1 vs _0_	**0.77 (0.66,	0.96 (0.92,	0.89 (0.88,**

**0.90)	0.99)	0.90)**

Hospital admissions: 2 vs 0	**0.73 (0.59,**	0.94 (0.88, 1.00)	**0.75 (0.72,**

**0.89)	0.77)**

Hospital admissions: ≥3 vs 0	1.04 (0.97, 1.12)	**0.50 (0.48,**

0.83 (0.67, 1.03)	**0.53)**

Comorbidities: COPD	**0.82 (0.68,**	0.96 (0.83, 1.12)	**0.67 (0.64,**

**1.00)	0.69)**

Comorbidities: hypertension	1.06 (0.92, 1.22)	**1.09 (1.04,	1.08 (1.06,**

**1.13)	1.09)**

Comorbidities: diabetes	1.12 (0.95, 1.31)	**1.27 (1.22,	1.31 (1.29,**

**1.33)	1.33)**

CIMD Residential instability: 2 vs 1	**0.73 (0.70,	0.94 (0.93,**

0.91 (0.78, 1.07)	**0.76)	0.95)**

CIMD Residential instability: 3 vs 1	**0.67 (0.65,	0.84 (0.82,**

1.09 (0.93, 1.28)	**0.70)	0.85)**

CIMD Residential instability: 4 vs 1	**0.59 (0.56,	0.67 (0.66,**

1.06 (0.89, 1.28)	**0.61)	0.69)**

CIMD Residential instability: 5 vs 1	**0.53 (0.51,	0.63 (0.62,**

0.98 (0.78, 1.24)	**0.55)	0.64)**

CIMD Economic dependency: 2 vs _1_	**1.60 (1.25,	0.92 (0.89,	1.06 (1.05,**

**2.05)	0.96)	1.07)**

CIMD Economic dependency: 3 vs 1	**1.91 (1.51,**	1.02 (0.98, 1.06)	0.99 (0.98, 1.01)

**2.42)**

CIMD Economic dependency: 4 vs 1	**2.55 (2.02,	1.06 (1.02,**	1.00 (0.99, 1.01)

**3.23)	1.10)**

CIMD Economic dependency: 5 vs _1_	**2.04 (1.60,	0.88 (0.84,	0.91 (0.89,**

**2.59)	0.92)	0.92)**

CIMD Ethnocultural composition: 2 vs 1	**0.85 (0.76,	1.16 (1.11,	1.18 (1.14,**

**0.95)	1.22)	1.21)**

**1.48 (1.41,	1.64 (1.59,**

CIMD Ethnocultural composition: 3 vs 1	1.11 (0.94, 1.30)	**1.56)	1.68)**

CIMD Ethnocultural composition: 4 vs 1	**1.54 (1.46,	2.50 (2.43,**

1.01 (0.80, 1.28)	**1.62)	2.57)**

CIMD Ethnocultural composition: 5 vs 1	**1.73 (1.64,	4.85 (4.72,**

1.15 (0.80, 1.65)	**1.82)	4.98)**

CIMD Situational vulnerability: 2 vs 1	0.85 (0.69, 1.04)	1.04 (0.99, 1.09)	**1.16 (1.15,**

**1.18)**

CIMD Situational vulnerability: 3 vs 1	**1.29 (1.05,	1.21 (1.14,	1.20 (1.18,**

**1.58)	1.28)	1.21)**

CIMD Situational vulnerability: 4 vs 1	**1.60 (1.32,	1.23 (1.16,	1.20 (1.18,**

**1.95)	1.30)	1.22)**

CIMD Situational vulnerability: 5 vs 1	**1.29 (1.04,	1.38 (1.30,	1.35 (1.33,**

**1.61)	1.47)	1.38)**

220 Adjusted odds ratios and 95% CIs are presented. The adjusted model contains all variables
221 listed in the table and does not contain additional covariates that are not listed in the table.

222

223 Having more than one hospitalization in the 3 years prior to the start of the study increased the
224 odds of being tested (Table 2, Figure 2). Conversely, having at least one hospitalization
225 corresponded to a decreased likelihood of testing positive (Table 3, Figure 3). In all provinces,
226 COPD, hypertension and diabetes were associated with a higher rate of testing (Table 2, Figure
227 2). Hypertension and diabetes also increased the odds of testing positive for SARS-CoV-2
228 (Table 3, Figure 3). Interestingly, having COPD conferred a lower likelihood of testing positive.
229 For provinces for which data was available, cancer, asthma, heart disease and dementia/frailty
230 were associated with increased SARS-CoV-2 testing (Supplemental Table 2). Cancer, asthma
231 and heart disease were associated with decreased odds of testing positive (Supplemental Table
232 3).
233 Residing in higher income areas was positively associated with testing in New Brunswick and
234 Ontario, but negatively associated with testing in Manitoba (Table 2, Figure 2). Higher
235 neighbourhood income was associated with significantly lower test positivity in Manitoba and
236 Ontario (Table 3, Figure 3).
237 Areas with higher ethnocultural composition exhibited lower testing rates (Table 2, Figure 2).
238 Both high ethnocultural composition and high situational vulnerability were associated with
239 increased odds of testing positive, whereas residential instability was associated with lower test
240 positivity (Table 3, Figure 3).

241

242 **Discussion**
243 In this work we sought to examine differences between SARS-CoV-2 testing and test positivity
244 rates between three Canadian provinces, as well as the demographic, medical history, SES and
245 SDH variables that may contribute to such differences. We observed that both testing rates and
246 test positivity rates varied significantly among provinces.
247 The COVID-19 pandemic affected Canadian provinces disparately and saw differing
248 governmental strategies to contain it. New Brunswick was part of the “Atlantic Bubble”, a group
249 of 4 provinces that severely restricted travel external to the bubble during the pandemic, while
250 Manitoba and Ontario had less severe restrictions. This likely decreased the burden of COVID-
251 19 in New Brunswick during the first three waves of the pandemic. Additionally, there exist
252 significant differences between the provinces’ distributions of population place of residence and
253 socio-ethnocultural composition, factors which we found to be important for determining SARS-
254 CoV-2 testing and test positivity.
255 We examined the possible confounding effects of pre-existing health status and comorbid
256 conditions on SARS-CoV-2 testing and positivity. COPD was associated with higher testing
257 rates and lower test positivity rates. One possible explanation for this finding is that COPD has
258 symptoms that mimic SARS-CoV-2 infection and may prompt SARS-CoV-2 testing more
259 frequently. Diabetes and hypertension have both been linked to developing serious illness due to
260 SARS-CoV-2 infection.^17,18^ Here we observed that the association between these comorbidities
261 and COVID-19 is reflected in higher test positivity rates. This may be attributable to an interplay
262 of factors, including immune dysfunction due to hyperglycemia in diabetic patients^19^ and
263 increase of the SARS-CoV-2 binding protein ACE2 in patients with hypertension or diabetes that
264 are treated with ACE inhibitors and/or angiotensin II type-1 receptor blockers.^20^
265 Place of residence was found to play an important role in SARS-CoV-2 testing, with urban
266 residents being more likely to be tested in all three provinces. This parallels what is previously
267 known about healthcare testing and capacity for other health conditions and has been
268 demonstrated for SARS-CoV-2 testing by Huang and colleagues.^21^ Reasons likely include
269 resource constraints that are magnified in rural areas, difficulty with transportation and cultural
270 perceptions of health and healthcare.^22-24^ As a significantly higher proportion of Ontario
271 residents are urban-dwellers (85%) compared to the other two provinces, place of residence is an
272 important contributor to the observed inter-province differences in testing. Interestingly, test
273 positivity effects were less consistent among provinces – urban residents tested positive more
274 frequently in Ontario and New Brunswick, but less frequently in Manitoba. This discrepancy
275 may reflect a testing drive that was done after an early outbreak in a rural northern area of
276 Manitoba.^25^ Differences in SARS-CoV-2 positivity between urban and rural areas could be
277 explained by factors associated with population density,^15,26^ such as differing levels of
278 congestion in living conditions, occupational exposures, use of public transportation, and
279 opportunity for social distancing.
280 Socioeconomic factors and SDH were found to play a significant role in SARS-CoV-2 testing
281 and positivity. Higher income was correlated with higher rates of testing in Ontario and New
282 Brunswick, while Manitoba was an outlier with highest rates of testing seen in the groups with
283 the lowest SES. This may relate to the testing drive in Northern Manitoba referenced earlier,
284 thus demonstrating the effectiveness of an equity-driven approach to testing in marginalized
285 communities. The observed dependence of SARS-CoV-2 testing on income is an area of
286 concern, as it may be a product of inequity in resource distribution and testing accessibility. Lack
287 of access to SARS-CoV-2 testing early in the disease course in highly impacted populations is
288 known to contribute to poorer health outcomes.^27^
289 Ethnocultural composition showed the strongest association to SARS-CoV-2 testing and testing
290 outcomes out of the five CIMD index components, with more racialized or higher immigrant rate
291 communities showing significantly lower testing rates and higher positivity rates. These
292 disparities are consistent with previous observations from Canada^15,28^ and the United States^29^ and
293 may stem from barriers to access to testing, institutionalized and systemic racism in health care,
294 differences in housing conditions, underlying health conditions or differing occupational
295 exposure risks.^30,31^ In Manitoba, 70% of cases to date have been reported to occur in First
296 Nations communities, although these communities make up only 10% of the population.^32^ Our
297 findings reinforce the urgent need to investigate contributors to barriers to testing in
298 ethnoculturally diverse communities and to devise community-specific pandemic response
299 planning strategies at the level of government and health policy makers.
300 Our study has important limitations. First, we were limited to cases confirmed in laboratory
301 testing and could not assess undiagnosed or rapid test-confirmed SARS-CoV-2 infections,
302 though for the period discussed here laboratory testing was employed most often. Income
303 quintile and social determinants of health were assessed at the neighborhood rather than at the
304 individual level, due to privacy limitations in data availability from Statistics Canada. Finally,
305 provinces differed in their testing strategies during the pandemic and experienced “waves” of
306 COVID-19 infection differently, which may have contributed to variability in the aggregate data.
307 **Conclusions**
308 In this study, we identified a series of demographic, medical, SES, and SDH factors that were
309 correlated with disparities in SARS-CoV-2 testing rates and test positivity. While highlighted by
310 the COVID-19 pandemic, such disparities may reflect underlying barriers to health care access
311 and utilization. Addressing COVID-19 testing inequity in at-risk groups (rural residents, high
312 ethnocultural composition, and lower income) requires unique and dedicated public health
313 initiatives.

314

315

316 **References:**
317 1. Caul S. Deaths involving COVID-19 by local area and socioeconomic deprivation: deaths occurring between 1
318 March and 31 July 2020. UK Office for National Statistics. See https://[www.](http://www/) ons. gov.
319 uk/peoplepopulationandcommunity/birthsdeathsandmarriages/deaths/bulletins/deathsinvolvingcovid19bylocalareasa
320 nddeprivation/deathsoccurringbetween1marchand31july2020. 2020 Aug 28.
321 2. Hull SA, Williams C, Ashworth M, Carvalho C, Boomla K. Prevalence of suspected COVID-19 infection in
322 patients from ethnic minority populations: a cross-sectional study in primary care. British Journal of General
323 Practice. 2020 Oct 1;70(699):e696-704.
324 3. Hawkins RB, Charles EJ, Mehaffey JH. Socio-economic status and COVID-19-related cases and fatalities. Public
325 Health. 2020 Dec;189:129-134. doi: 10.1016/j.puhe.2020.09.016. Epub 2020 Oct 17. PMID: 33227595.
326 4. Dorn AV, Cooney RE, Sabin ML. COVID-19 exacerbating inequalities in the US. Lancet. 2020 Apr
327 18;395(10232):1243-1244. doi: 10.1016/S0140-6736(20)30893-X. PMID: 32305087; PMCID: PMC7162639.
328 5. Lamb MR, Kandula S, Shaman J. Differential COVID-19 case positivity in New York City neighborhoods:
329 Socioeconomic factors and mobility. Influenza Other Respir Viruses. 2021 Mar;15(2):209-217. doi:
330 10.1111/irv.12816. Epub 2020 Oct 14. PMID: 33280263; PMCID: PMC7675704.
331 6. Mackey K, Ayers CK, Kondo KK, Saha S, Advani SM, Young S, Spencer H, Rusek M, Anderson J, Veazie S,
332 Smith M, Kansagara D. Racial and Ethnic Disparities in COVID-19-Related Infections, Hospitalizations, and Deaths
333 : A Systematic Review. Ann Intern Med. 2021 Mar;174(3):362-373. doi: 10.7326/M20-6306. Epub 2020 Dec 1.
334 PMID: 33253040; PMCID: PMC7772883.
335 7. Abedi V, Olulana O, Avula V, Chaudhary D, Khan A, Shahjouei S, Li J, Zand R. Racial, Economic and Health
336 Inequality and COVID-19 Infection in the United States. medRxiv [Preprint]. 2020 May 1:2020.04.26.20079756.
337 doi: 10.1101/2020.04.26.20079756. Update in: J Racial Ethn Health Disparities. 2020 Sep 1;: PMID: 32511647;
338 PMCID: PMC7277003.
339 8. Khanijahani A, Iezadi S, Gholipour K, Azami-Aghdash S, Naghibi D. A systematic review of racial/ethnic and
340 socioeconomic disparities in COVID-19. Int J Equity Health. 2021 Nov 24;20(1):248. doi: 10.1186/s12939-021-
341 01582-4. PMID: 34819081; PMCID: PMC8611382.
342 9. Lyng GD, Sheils NE, Kennedy CJ, Griffin DO, Berke EM. Identifying optimal COVID-19 testing strategies for
343 schools and businesses: Balancing testing frequency, individual test technology, and cost. PLoS ONE. 2021; 16(3):
344 e0248783[.](https://doi.org/10.1371/journal.pone.0248783) <https://doi.org/10.1371/journal.pone.0248783>
345 10. Lee, D., and Lee, J. Testing on the move: South Korea's rapid response to the COVID-19 pandemic. Transport.
346 Res. Interdiscip. Perspect. 2020; 5:100111. doi: 10.1016/j.trip.2020.100111
347 11. Ali S, Asaria M, Stranges S. COVID-19 and inequality: are we all in this together? Can J Public Health. 2020
348 Jun;111(3):415-416. doi: 10.17269/s41997-020-00351-0. Epub 2020 Jun 23. PMID: 32578185; PMCID:
349 PMC7310590.
350 12. Gao J, Quan L. Current Status of Diagnostic Testing for SARS-CoV-2 Infection and Future Developments: A
351 Review. Med Sci Monit. 2020 Dec 17;26:e928552. doi: 10.12659/MSM.928552. PMID: 33332288; PMCID:
352 PMC7754691.
353 13. Lieberman-Cribbin, W., Alpert, N., Flores, R. *et al.* Analyzing disparities in COVID-19 testing trends according
354 to risk for COVID-19 severity across New York City. BMC Public Health. 2021; 21, 1717[.](https://doi.org/10.1186/s12889-021-11762-0)
355 <https://doi.org/10.1186/s12889-021-11762-0>
356 14. Seto E, Min E, Ingram C, Cummings BJ, Farquhar SA. Community-Level Factors Associated with COVID-19
357 Cases and Testing Equity in King County, Washington. Int J Environ Res Public Health. 2020 Dec 18;17(24):9516.
358 doi: 10.3390/ijerph17249516. PMID: 33353095; PMCID: PMC7767300.*(London, England)*, *395*(10229), 1054–
359 1062.
360 15. Sundaram ME, Calzavara A, Mishra S, Kustra R, Chan AK, Hamilton MA, Djebli M, Rosella LC, Watson T,
361 Chen H, Chen B, Baral SD, Kwong JC. Individual and social determinants of SARS-CoV-2 testing and positivity in
362 Ontario, Canada: a population-wide study. CMAJ. 2021 May 17;193(20):E723-E734. doi: 10.1503/cmaj.202608.
363 Epub 2021 Apr 27. PMID: 33906966; PMCID: WangPMC8177943.
364 16. [Canadian Index of Multiple Deprivation: User Guide:](https://www150.statcan.gc.ca/n1/pub/45-20-0001/452000012019002-eng.htm) [https://www150.statcan.gc.ca/n1/pub/45-20-](https://www150.statcan.gc.ca/n1/pub/45-20-0001/452000012019002-eng.htm)
365 [0001/452000012019002-eng.htm](https://www150.statcan.gc.ca/n1/pub/45-20-0001/452000012019002-eng.htm)
366 17. Wang D, Hu B, Hu C, Zhu F, Liu X, Zhang J, Wang B, Xiang H, Cheng Z, Xiong Y, Zhao Y, Li Y, Wang X,
367 Peng Z. Clinical Characteristics of 138 Hospitalized Patients With 2019 Novel Coronavirus-Infected Pneumonia in
368 Wuhan, China. JAMA. 2020 Mar 17;323(11):1061-1069.
369 18. Zhou, F., Yu, T., Du, R., Fan, G., Liu, Y., Liu, Z., Xiang, J., Wang, Y., Song, B., Gu, X., Guan, L., Wei, Y., Li,
370 H., Wu, X., Xu, J., Tu, S., Zhang, Y., Chen, H., & Cao, B. Clinical course and risk factors for mortality of adult
371 inpatients with COVID-19 in Wuhan, China: a retrospective cohort study. Lancet. 2020; PMC7568122.
372 19. Landstra CP and de Koning EJP. COVID-19 and diabetes: understanding the interrelationship and risks for a
373 severe course. Front. Endocrinol. 2021; 12:649525.
374 20. Fang L, Karakiulakis G, Roth M. Are patients with hypertension and diabetes mellitus at increased risk for
375 COVID-19 infection? Lancet Respir Med. 2020 Apr;8(4):e21.
376 21. Huang Q, Jackson S, Derakhshan S, Lee L, Pham E, Jackson A, Cutter SL. Urban-rural differences in COVID-
377 19 exposures and outcomes in the South: A preliminary analysis of South Carolina. PLoS One. 2021 Feb
378 3;16(2):e0246548. doi: 10.1371/journal.pone.0246548. PMID: 33534870; PMCID: PMC7857563.
379 22. Anderson TJ, Saman DM, Lipsky MS, Lutfiyya MN. A cross-sectional study on health differences between rural
380 and non-rural U.S. counties using the County Health Rankings. BMC Health Serv Res. 2015 Oct 1;15:441. doi:
381 10.1186/s12913-015-1053-3. PMID: 26423746; PMCID: PMC4590732.
382 23. Arcury TA, Preisser JS, Gesler WM, Powers JM. Access to transportation and health care utilization in a rural
383 region. J Rural Health. 2005 Jan;21(1):31–8. pmid:15667007
384 24. Douthit N, Kiv S, Dwolatzky T, Biswas S. Exposing some important barriers to health care access in the rural
385 USA. Public Health. 2015 Jun;129(6):611–20. pmid:26025176
386 25. News release, Government of Manitoba: “Additional COVID-19 testing to offer rapid results for
387 Northern Manitoba Patients”. Can be found at: https://news.gov.mb.ca/news/?archive=&item=48438.
388 26. Rideout A, Murray C, Isles C. Regional variation in COVID-19 positive hospitalization across Scotland during
389 the first wave of the pandemic and its relation to population density: A cross-sectional observation study. PLoS One.
390 2021 Jul 9;16(7):e0253636. doi: 10.1371/journal.pone.0253636. PMID: 34242268; PMCID: PMC8270435.
391 27. Chen JT, Krieger N. Revealing the Unequal Burden of COVID-19 by Income, Race/Ethnicity, and Household
392 Crowding: US County Versus Zip Code Analyses. J Public Health Manag Pract. 2021 Jan/Feb;27 Suppl 1, COVID-
393 19 and Public Health: Looking Back, Moving Forward:S43-S56. doi: 10.1097/PHH.0000000000001263. PMID:
394 32956299.
395 28. Campbell-Scherer D, Chiu Y, Ofosu NN, Luig T, Hunter KH, Jabbour B, Farooq S, Mahdi A, Gayawira A,
396 Awasis F, Olokude F, Goa H, Syed H, Sillito J, Yip L, Belle L, Akot M, Nutter M, Farhat N, Wang Y, Jalal N,
397 Khalif S, Chapagain S, Fernandez S, Azarcon S, Hama Z. Illuminating and mitigating the evolving impacts of
398 COVID-19 on ethnocultural communities: a participatory action mixed-methods study. CMAJ. 2021 Aug
399 9;193(31):E1203-E1212. doi: 10.1503/cmaj.210131. PMID: 34373268; PMCID: PMC8367425.
400 29. Tai, D., Shah, A., Doubeni, C. A., Sia, I. G., & Wieland, M. L. The Disproportionate Impact of COVID-19 on
401 Racial and Ethnic Minorities in the United States. Clinical infectious diseases : an official publication of the
402 Infectious Diseases Society of America. 2021; *72*(4), 703–706. <https://doi.org/10.1093/cid/ciaa815>
403 30. Mishra S, Ma H, Moloney G, Yiu KCY, Darvin D, Landsman D, Kwong JC, Calzavara A, Straus S, Chan AK,
404 Gournis E, Rilkoff H, Xia Y, Katz A, Williamson T, Malikov K, Kustra R, Maheu-Giroux M, Sander B, Baral SD;
405 COVID-19 Heterogeneity Research Group. Increasing concentration of COVID-19 by socioeconomic determinants
406 and geography in Toronto, Canada: an observational study. Ann Epidemiol. 2022 Jan;65:84-92. doi:
407 10.1016/j.annepidem.2021.07.007. Epub 2021 Jul 25. PMID: 34320380; PMCID: PMC8730782.
408 31. Buchan SA, Smith PM, Warren C*, et al*Incidence of outbreak-associated COVID-19 cases by industry in
409 Ontario, Canada, 1 April 2020–31 March 2021. Occupational and Environmental Medicine. 2022;**79:**403-411.
410 32. Emily Cameron-Blake, Charles Breton, Paisley Sim, Helen Tatlow, Thomas Hale, Andrew Wood, Jonathan
411 Smith, Julia Sawatsky, Zachary Parsons, Katherine Tyson (2021). “Variation in the Canadian Provincial and
412 Territorial responses to COVID19”. Blavatnik School of Government Working Paper. Available:
413 [www.bsg.ox.ac.uk/covidtracker](http://www.bsg.ox.ac.uk/covidtracker)

414

415

416

417

418

419

420

421

422

# 423 Supporting Information

424

425	**S1 Supplemental Table 1:** Database data sources for the provinces of New Brunswick (NB),

426	Manitoba (MB) and Ontario (ON).

427

428	**S2 Supplemental Table 2:** Odds ratios and confidence intervals for multivariable regression

429	models of the odds of being tested for SARS-CoV-2, in New Brunswick (NB), Manitoba (MB),

430	and Ontario (ON), including additional covariates for comorbidities and air pollution.

431

432	**S3 Supplemental Table 3:** Odds ratios and confidence intervals for multivariable regression

433	models of the odds of testing positive for SARS-CoV-2 among tested individuals, in New

434	Brunswick (NB), Manitoba (MB), and Ontario (ON), including additional covariates for

435	comorbidities and air pollution.

436

437	**S4 Supplemental Table 4:** Odds ratios and confidence limits for univariate regression models of

438	SARS-CoV-2 tested individuals in New Brunswick (NB), Manitoba (MB), and Ontario (ON).

439	Bolded values indicate significance.

440

441	**S5 Supplemental Table 5:** Odds ratios and confidence limits for univariate regression models

442	of individuals testing positive for SARS-CoV-2 in New Brunswick (NB), Manitoba (MB), and

443	Ontario (ON). Bolded values indicate significance.

444

445

446

447

# 448 Authors' Contributions

449 All co-authors made substantial contributions to this manuscript. Specifically: MC and SJO were
450 most responsible for conceptualization, funding acquisition, methodology, project
451 administration, supervision, original draft preparation and manuscript review; LA was
452 responsible for data verification and interpretation, project administration, formal analysis,
453 original draft preparation and critical review and editing of the manuscript; CS, MSirski and
454 MSundaram were responsible for investigation, data curation and formal analysis; JC was
455 responsible for data curation and visualization; JTM, SM, JCK, AK, SB, LC, AC were
456 responsible for methodology, interpretation of the data, and review and editing of the
457 manuscript; all authors were responsible for final approval of the article.

458

459

<https://www.editorialmanager.com/pone/download.aspx?id=31851925&guid=4ad018ec-d2f0-4514-9556-925594b27046&scheme=1>Figure 1	[Click here to access/download;Figure;Figure 1 Sept 12 2022.docx](https://www.editorialmanager.com/pone/download.aspx?id=31851925&guid=4ad018ec-d2f0-4514-9556-925594b27046&scheme=1)

<https://www.editorialmanager.com/pone/download.aspx?id=31851926&guid=cdb1d0bc-cc62-4811-8086-107785f90f43&scheme=1>Figure 2	[Click here to access/download;Figure;Figure 2 Sept 12 2022.docx](https://www.editorialmanager.com/pone/download.aspx?id=31851926&guid=cdb1d0bc-cc62-4811-8086-107785f90f43&scheme=1)

<https://www.editorialmanager.com/pone/download.aspx?id=31851927&guid=c4b582b4-2942-4acd-a149-a5c24a37a98c&scheme=1>Figure 3	[Click here to access/download;Figure;Figure 3 Sept 12 2022.docx](https://www.editorialmanager.com/pone/download.aspx?id=31851927&guid=c4b582b4-2942-4acd-a149-a5c24a37a98c&scheme=1)

Supplemental Tables

[Click here to access/download](https://www.editorialmanager.com/pone/download.aspx?id=31851928&guid=ce974995-8609-4cb2-86f0-b5bdd7028b86&scheme=1)

[Supporting Information Supplemental Tables Sept 12 2022.docx](https://www.editorialmanager.com/pone/download.aspx?id=31851928&guid=ce974995-8609-4cb2-86f0-b5bdd7028b86&scheme=1)

Supplemental Table 1

[Click here to access/download](https://www.editorialmanager.com/pone/download.aspx?id=31851929&guid=4b5b6151-71de-41e2-ad2e-81fc46416169&scheme=1)

[Supporting Information](https://www.editorialmanager.com/pone/download.aspx?id=31851929&guid=4b5b6151-71de-41e2-ad2e-81fc46416169&scheme=1)

[S6 Supplemental Figure 1 Sept 12 2022.docx](https://www.editorialmanager.com/pone/download.aspx?id=31851929&guid=4b5b6151-71de-41e2-ad2e-81fc46416169&scheme=1)

Supplemental Table 2

[Click here to access/download](https://www.editorialmanager.com/pone/download.aspx?id=31851930&guid=2d46770c-b62a-4025-8b15-9630f53181c8&scheme=1)

[Supporting Information](https://www.editorialmanager.com/pone/download.aspx?id=31851930&guid=2d46770c-b62a-4025-8b15-9630f53181c8&scheme=1)

[S7 Supplemental Figure 2 Sept 12 2022.docx](https://www.editorialmanager.com/pone/download.aspx?id=31851930&guid=2d46770c-b62a-4025-8b15-9630f53181c8&scheme=1)
