# Supplementary material for: Comparison of socio-economic determinants of COVID-19 testing and positivity in Canada: A multi-provincial analysis
Source: PLoS One. 2023 Aug 23;18(8):e0289292. doi: 10.1371/journal.pone.0289292 (PMC10446177; doi:10.1371/journal.pone.0289292)
Supplement: Attachment — Submitted filename: Response to reviewers Dec 03 2022.docx [file pone.0289292.s009.docx]

## PLOS ONE Decision: Revision required [PONE-D-22-25812] - [EMID:73fa18e816edcb89]

[em.pone.a.7ea](mailto:em.pone.a.7ea175.3fcd2437@editorialmanager.com)[175.3fcd2437@editorialmanager.com](mailto:175.3fcd2437@editorialmanager.com)

<em.pone [.0.7ea175.3fcd2437@editorialmanager.com](mailto:.0.7ea175.3fcd2437@editorialmanager.com)> on behalf of

PLOS ONE [<em@editorialmanager.com>](mailto:em@editorialmanager.com)

Wed 2022-10-12 12:01 PM

To: Lilia Antono va <lantono [va@ohri.ca](mailto:va@ohri.ca) >

 1 att achment s (408 KB)

PONE-D-22-25812_reviewer1.docx;

**CAUTION: External Mail.** Do not click on links or open attachments you do not trust.

**ATTENTION: Courriel externe.** Ne cliquez pas sur des liens et n'ouvrez pas de pieces jointes auxquelles vous ne faites pas confiance.

PONE-D-22-25812

Comparison of socio-economic determinants of COVID-19 testing and positivity in Canada: a multi-provincial analysis .

PLOS ONE

Dear Dr. Antonova,

Thank you for submitting your manuscript to PLOS ONE. After careful consideration, we feel that it has merit but does not fully meet PLOS ONE' s publication criteria as it currently sta nds. Therefore, we invite you to submit a revised version of the manuscript that addresses the points raised during the review process.

Thank you very much for the opportunity. We have carefully considered and addressed all reviewer comments.

ACADEMIC EDITOR:

Please address all of the reviewer's comments.

This has been done.

Play particular attention to explaining the study design, and mentioning that three separate analysis was undertaken for each Province.

The reviewer comments have been addressed and this has been clarified in the text.

Please describe the statistical approach used, and I would suggest to use a single multivariable regression model instead of several univariable models.

We have clarified that the results in Tables 2 and 3 are from a multivariable regression model and the model has been added into Materials and Methods.

Please submit your revised manuscript by Nov 26 2022 11:59PM. If you will need more time than this to complete your revisions, please reply to this message or contact the journal office at [plosone@plos.o rg.](mailto:plosone@plos.org) When you're ready to submit your revision, log on to [https://www.editorialmanager.com/pone/](http://www.editorialmanager.com/pone/) and select the 'Submissi ons Needing Revision' folder to locate your manuscript file.

Please include the following items when submitting your revised manuscript:

- A rebuttal letter that responds to each point raised by the academic editor and reviewer(s). You should upload this letter as a separate file labeled 'Response to Reviewers'.
- A marked-up copy of your manuscript that highlights changes made to the original version. You should upload this as a separate file labeled 'Revised Manuscript with Track Changes'.
- An unmarked version of your revised paper without tracked changes. You should upload this as a separate file labeled 'Manu script'.

If you would like to make changes to your financial disclosure, please include your updated statement in your cover letter . Guidelines for resubmitting your figure files are available below the reviewer comments at the end of this letter.

If applicable, we recommend that you deposit your laboratory protocols in protocols.i o to enhance the reproducibility of your results. Protocols.io assigns your protocol its own identifier (DOI) so that it can be cited independently in the future. For instructions see: http s://jo urnals.plos.org/ plosone/s/ submissi on-guidelines#l oc­ laborat ory-p rotocols. Additionally, PLOS ONE offers an option for publishing peer-reviewed Lab Protocol articles, which describe protocols hosted on protocols.io . Read more information on sharing protocols at https://plos.org/protocols?utm medium=editorial-email&utm source=authorletters&utm campaign=protocols.

We look forward to receiving your revised manuscript. Kind regards,

Csaba Varga, DVM MSc PhD Academic Editor

PLOS ONE

Journal Requirement s:

https://outlook.office.com/mail/inbox/id/AAQkAGEzMGNhZGQwLTMwNmYtNDMyYiyM1zY3LTUxNTFIYWQxZDNiZQAQANx%2B5mckxUFEmvxvOw	1/5

10/21/22, 10:39 AM	Mail - Lilia Antonova - Outlook

# When submitting your revision, we need you to address these additional requirements.

1. Please ensure that your manuscript meets PLOS ONE's style requirements, including those for file naming. The PLOS ONE style templates can be found at https://journals.plos.org/plosone/s/file?id=wjVg/PLOSOne_formatting_sample_main_body.pdf and https://journals.plos.org/plosone/s/file?id=ba62/PLOSOne_formatting_sample_title_authors_affiliations.pdf
2. Please ensure that you have specified (1) whether consent was informed and (2) what type you obtained (for instance, written or verbal, and if verbal, how it was documented and witnessed). If your study included minors, state whether you obtained consent from parents or guardians. If the need for consent was waived by the ethics committee, please include this information.

As this was a retrospective study using aggregate Health Administrative Database data, it does not involve patient consent. The ethics board approval information has been put into a separate sub-section within Materials and Methods for clarity and as requested in reviewer comments.

3. We note that the grant information you provided in the 'Funding Information' and 'Financial Disclosure' sections do not match.

When you resubmit, please ensure that you provide the correct grant numbers for the awards you received for your study in the 'Funding Information' section.

No specific awards were received for this study. Any support listed in the funding statement was provided through data access only on individual investigator support for COVID-19 related research, but not specifically this work. The funders had no role in study design, data collection and analysis, decision to publish, or preparation of the manuscript.

4. Thank you for stating the following financial disclosure:

"Funding and Disclaimers

This work was supported by Public Health Ontario. This study was also supported by ICES, which is funded by an annual grant from the Ontario Ministry of Health (MOH) and the Ministry of Long-Term Care (MLTC). This study was supported by the Ontario Health Data Platform (OHDP), a Province of Ontario initiative to support Ontario's ongoing response to COVID-19 and its related impacts. The analyses for Ontario and Manitoba were supported by the Canadian Institutes of Health Research (VRS-172683). This work was supported by the Department of Health and Vitalite Health Network of the Province of New Brunswick under a contract with the New Brunswick Institute for Research, Data and Training at the University of New Brunswick. The results and conclusions are those of the authors and no official endorsement by the Government of New Brunswick or Vitalite Health Network was intended or should be inferred. The study sponsors did not participate in the design and conduct of the study; collection, management, analysis and interpretation of the data; preparation, review or approval of the manuscript; or the decision to submit the manuscript for publication. Parts of this material are based on data and/or information compiled and provided by the Canadian Institute for Health Information (CIHI) and by Ontario Health (OH). However, the analyses, conclusions, opinions and statements expressed herein are solely those of the authors, and do not reflect those of the funding or data sources; no endorsement by ICES, MOH, MLTC, OHDP, its partners, the Province of Ontario, CIHI or OH is intended or should be inferred. J.C.K. is supported by a Clinician-Scientist Award from the University of Toronto Department of Family and Community Medicine. SM is supported by a Tier 2 Canada Research Chair in Mathematical Modeling and Program Science."

Please state what role the funders took in the study. If the funders had no role, please state: "The funders had no role in study design, data collection and analysis, decision to publish, or preparation of the manuscript."

If this statement is not correct you must amend it as needed.

Please include this amended Role of Funder statement in your cover letter; we will change the online submission form on your behalf.

The funders had no role in study design, data collection and analysis, decision to publish, or preparation of the manuscript.

5. In your Data Availability statement, you have not specified where the minimal data set underlying the results described in your manuscript can be found. PLOS defines a study's minimal data set as the underlying data used to reach the conclusions drawn in the manuscript and any additional data required to replicate the reported study findings in their entirety. All PLOS journals require that the minimal data set be made fully available. For more information about our data policy, please see [http://journals.plos.org/plosone/s/data-availability.](http://journals.plos.org/plosone/s/data-availability)

"Upon re-submitting your revised manuscript, please upload your study's minimal underlying data set as either Supporting Information files or to a stable, public repository and include the relevant URLs, DOis, or accession numbers within your revised cover letter. For a list of acceptable repositories, please see [http://journals.plos.org/plosone/s/data-availability#loc-recommended-repositories.](http://journals.plos.org/plosone/s/data-availability#loc-recommended-repositories) Any potentially identifying patient information must be fully anonymized.

Important: If there are ethical or legal restrictions to sharing your data publicly, please explain these restrictions in detail. Please see our guidelines for more information on what we consider unacceptable restrictions to publicly sharing data: [http://journals.plos.org/plosone/s/data-availability#loc-unacceptable-data-access­](http://journals.plos.org/plosone/s/data-availability#loc-unacceptable-data-access) restrictions. Note that it is not acceptable for the authors to be the sole named individuals responsible for ensuring data access.

We will update your Data Availability statement to reflect the information you provide in your cover letter.

The dataset from this study is held securely in coded form at provincial HADs. While legal data sharing agreements between HADs and data providers (e.g., healthcare organizations and government) prohibit the respective HADs from making the dataset publicly available, access may be granted to those who meet prespecified criteria for confidential access (for the province of Ontario available at [www.ices.on.ca/DAS](http://www.ices.on.ca/DAS) [email: das@ices.on.ca]). The full dataset creation plan and underlying analytic code are available from the authors upon request, understanding that the computer programs may rely upon coding templates or macros that are unique to the HADs and are therefore either inaccessible or may require modification.

6. Please include a copy of Table 1 which you refer to in your text on page 8.

Apologies. It appears that while Table 1 was included in the manuscript, the Table 1 title was omitted. We have now added the title for Table 1.

7. Please remove your figures from within your manuscript file, leaving only the individual TIFF/EPS image files, uploaded separately. These will be automatically included in the reviewers' PDF.

This has been done.

8. Please review your reference list to ensure that it is complete and correct. If you have cited papers that have been retracted, please include the rationale for doing so in the manuscript text, or remove these references and

replace them with relevant current references. Any changes to the reference list should be mentioned in the rebuttal letter that accompanies your revised manuscript. If you need to cite a retracted article, indicate the article's retracted status in the References list and also include a citation and full reference for the retraction notice.

This has been done.

**Reviewer #1:** This manuscript examines the differences in COVID-19 testing and likelihood of testing positive for COVID-19 across numerous demographic and social factors in three Canadian provinces. It is extremely well written, clear, concise, yet rigorous in the statistical analysis. I believe it is almost ready for publication consideration after addressing the following:

-I am surprised that the 20-49 age group were most likely to be tested considering the highest risk of severe disease and death among the elderly. Is this associated with limited mobility, access to care, etc? I wonder if you should have included those in long-term care homes (but of course, that could be a separate study...).

Long-term care residents in Canada constitute a unique population in terms of their access to SARS-CoV-2 testing and vaccination in the course of the pandemic. Due to frequent COVID-19 outbreaks in long-term care homes, residents have been tested significantly more frequently in the course of the pandemic. In addition to being given access to frequent testing, long-term care residents were given highest vaccination priority, leading to an estimated 95% vaccination rate by March 31st 2021, as compared to only 3% of the general population. (Public Health Agency of Canada. COVID-19 vaccination in Canada. https://health-infobase.canada.ca/covid-19/vaccination-coverage/). These factors indicate that SARS-CoV-2 testing and positivity in long-term care residents are likely to be fundamentally different than those in the general population. Therefore, the inclusion of long-term care residents in the study would have likely artificially skewed the age-adjusted results, which is the reason that they were excluded from the study. To clarify this in the manuscript, the following text was added to lines 139 and 140 on page 6: “Long-term care residents represent a population with unique access to testing and vaccination in the course of the pandemic, which precludes their comparison to the general population.”

-Did you consider adjusting for time since testing effort and availability will have increased substantially since the start of the pandemic (which is the start of your study period)?

While we had originally considered performing an analysis for different time periods for the different provinces, upon consultation among provincial coinvestigators, we decided against it for the following reasons:

-Covid-19 waves did not occur at the same time in the different provinces and were substantially delayed in the Atlantic provinces (New Brunswick). This is also the case for vaccine roll-outs. Therefore, we would have observed large differences within time periods for testing and positivity, which would have been due to the differential arrival of Covid-19 and would have not informed the main research questions in this work, while precluding the understanding of overall trends across provinces.

-The focus of this work was on the effect of social determinants of health on Covid-19 testing and positivity. In order to focus on the effect of the variables of interest, we needed to be able to control for other factors, including short-term differences in infection rates across provinces. Therefore, assessment of the overall time period was deemed most appropriate for our research purposes.

-I think it is important to show time series of testing and positivity rates for the 3 provinces.

Addressed in the response to the previous comment.

-Do you suspect these significant relationships may have changed after April of 2021 due to vaccine rollouts, increased access to testing, and the Omicron surges? Why exclude more recent data? Please discuss.

In this work we have included the most recent data available to date at the time of commencement of data analysis. Importantly, the arrival of Omicron in Canada closely coincided with the roll-out of antigen rapid testing, resulting in a significant shift away from PCR testing and towards rapid testing, the results of which are not centrally reported. Therefore, this study would not be possible for data obtained much later than the arrival of Omicron. In this work, we have employed the unique opportunity, available only for a certain period in time, of widely available and universally reported testing results for a pandemic in progress, as a prism through which we can identify vulnerable populations and inequities in healthcare access.

To clarify, we have added in Materials and Methods: “This (study) period also precedes the shift towards antigen rapid-testing, the results of which are not collected in provincial healthcare databases.” We have also added to the beginning of Discussion: “In this work we accessed the unique opportunity of having systematically recorded testing and test result data for a pandemic in progress in order to identify disparities in socioeconomic vulnerability to health emergencies and inequities in access to healthcare.”

-Can you provide pseudo R squared values for the multivariate models?

Thank you for the suggestion however there is no standard acceptable version of R-squared to report for multivariable logistic regression. R-squared is primarily used for model comparison purposes. In our case, with an a priori model, as explained under Analysis, no model comparisons were performed.

-It is not clear if you ran numerous MV regression models or the results in Tables 2 and 3 are one big model, respectively, for each province. Furthermore, was any multicollinearity testing conducted? Might also be good to provide a correlation matrix of your variables.

We confirm that the results presented in Tables 2 and 3 are indeed multiple regression results, as specified in the table titles. These are the fully adjusted models. Therefore, for each dependent variable presented, all other variables reported in the table were included in the analysis as independent variables. Univariate regression analyses are included in the Supplemental data tables only. The logistic regression models are now included under Materials and Methods and the table legends specify that all variables in the respective table have been included in the multiple regression analysis. Additionally, we have added the term “independent” variable to the title of column 1 of the tables for clarity. Due to space constraints, we are not able to present both the unadjusted and adjusted results in the same table, which is why we have selected to present the multivariate results within the body of the manuscript and have included the univariate results under Supplemental data.

In the previous smaller-scale Ontario- based investigation carried out by this team, we had performed a collinearity investigation for all independent variables included in this current paper by using tolerances and inflation factors. No collinearity was detected for any of the variables and no variables had to be excluded.

We have added the logistic regression models into the Analysis section of Materials and Methods. We have also clarified that: “A priori covariate selection was based on previous research conducted by our team.^15^ Collinearity between the included variables was assessed in this previous investigation and was not found for any of the variables.”

-You should also discuss the opportunity to conduct spatial regression/analysis since you have a robust dataset that can be aggregated at the neighborhood level. Therefore, you can better identify spatial factors of testing and positivity.

This is an interesting question and we have looked at this in other provincial analyses that have already been published (ex.: Xia Y, Ma H, Moloney G, Velásquez García HA, Sirski M, Janjua NZ, Vickers D, Williamson T, Katz A, Yiu K, Kustra R, Buckeridge DL, Brisson M, Baral SD, Mishra S, Maheu-Giroux M. Geographic concentration of SARS-CoV-2 cases by social determinants of health in metropolitan areas in Canada: a cross-sectional study. CMAJ. 2022 Feb 14;194(6):E195-E204. doi: 10.1503/cmaj.211249. PMID: 35165131; PMCID: PMC8900797). However, in this study we looked at neighbourhood-level characteristics and how they affect our outcomes of interest. While clustering to geographical areas is a very interesting question, it was outside the scope of our investigation, as we were interested in assessing province-wide phenomena in order to compare them to other provinces.

Reviewer #2: Thank you for the opportunity to review this manuscript. Overall this work provides an interesting overview of various factors that may have impacted COVID testing and test positivity in NB, MB and ON. I think that this manuscript could be greatly strengthened by inclusion of additional information, specifically regarding why individual provinces were chosen for inclusion, and additional details regarding the model building process, sufficient for these analyses to be repeated. There appear to be some inconsistencies between model output presented in tables versus within text. Specific comments/suggestions are as follows:

Abstract:

- suggest clarifying within the methods section that data from each province were modelled separately as this is not evident until reading the full methods section.

We have added the following sentence to the abstract for clarity: “Data was modelled separately for each province in accordance to a prespecified protocol and follow-up consultations among provincial statisticians and collaborators.” and have specified that data was obtained from provincial health administrative databases.

- it is not clear in the abstract whether variables that were associated with testing or positivity were those from univariate or multivariate analyses - suggest clarifying (e.g., after adjustment for other variables, ...)

“After adjustment for other variables” has been added to the first sentence of the Results sub-section of the abstract for clarity.

- the results state that high ethnocultural composition was associated with lower testing, however per Table 2, this is only true across all provinces examined for the highest quintile compared to the lowest - avoid generalizations with respect to trend where results differ across quintiles.

This has been revised to “highest ethnocultural composition”.

Introduction:

- It is not clear why, when a similar analysis was already completed for Ontario (per Reference 15 - Sunderam et al.), this was largely repeated here rather than any similarities to the published study using Ontario data commented on within the discussion.

The purpose was to extend the study period from the prior Ontario study and to add other provinces to determine if the challenges in access to testing were different across provinces. The data set for this paper contains almost an entire year’s worth of additional data from Ontario, as well as data from the provinces of New Brunswick and Manitoba. This was clarified in the text as in the response to the comment below.

- Suggest adding additional context re: why this was repeated (particularly since supplementary tables contain variables included in the original study that are not mentioned or discussed anywhere in this manuscript and that were not included in analyses for other provinces) - presumably this was to allow for a detailed side by side comparison of model output for the different provinces

We have revised the last paragraph of the Introduction in order to provide context: “In order to assess COVID-19 healthcare access and vulnerability across Canada, we sought to add to previous Canadian findings^15^ by evaluating differences in SARS-CoV-2 testing rates and test results according to demographic, medical history, SES and SDH variables.”

We have also clarified under Materials and Methods the reason for including some additional variables on under Supplemental Data. “Variables for which data were not available in all provinces were not included in the main models in order to allow for side-to-side comparisons between provinces. However, they can be found in additional multiple regression analyses presented under Supplemental Data for reference.”

- It is not clear why MB and NB were chosen for compari son. Suggest adding some additional information regarding the choice of provinces compared - i.e., availability of comparable data, similarities with respect to access to testing, or by random selection of geographically dispersed provinces in Canada.

We had previously specified that: “We conducted a population-based, cross-sectional, observational study across three Canadian provinces (New Brunswick [NB], Manitoba [MB], and Ontario [ON]), which differ in their sizes and population distributions, as well as in the relative impact of COVID-19.” We have now added more detail to this section by specifying: “In particular, MB has a large Northern community outside of Winnipeg. NB on the other hand is largely rural, without an obvious ‘central hub’ city. Additionally, the Eastern Provinces had a different approach to minimizing the impact of COVID-19 on the community by imposing a travel ban to and from all other provinces, which produce a delay in the waves of COVID-19 and the associated strains on testing capacity.”

Methods:

- It is not clear why data were collected for some variables reported in either the methods or supplementary tables (e.g., comorbidities, air pollution) when these are not mentioned in the results/discussion. From the supplemental tables it appears that these were significantly associated with the outcomes of interest and inclusion of these in multivariate models changed the direction of association for some variables (e.g., the odds of individuals aged 85+ being tested relative to those aged 0-4 years).

As the goal was to assess trends across provinces, we did not want to include variables for which we do not have a complete interprovincial data set in the manuscript. However, we have included results for these additional variables under Supplemental Data for readers who are interested in these variables. We are happy to take these out of Supplemental Data if preferred.

We have clarified in Materials and Methods that: “Variables for which data were not available in all provinces were not included in the main models in order to allow for side-to-side comparisons between provinces. However, they can be found in additional multiple regression analyses presented under Supplemental Data for reference.”

- There is very little information provided regarding the data modelling approach used. Suggest adding additional information, e.g. if variables were assessed for collinearity, if a backwards approach was used for multivariate models, if model fit was checked.

In the previous Ontario- based investigation carried out by this team, we had performed a collinearity investigation for all independent variables included in this current paper by using tolerances and inflation factors. No collinearity was detected for any of the variables and no variables had to be excluded.

We have added the logistic regression models into the Analysis section of Materials and Methods. We have also clarified that: “A priori covariate selection was based on previous research conducted by our team.^15^ Collinearity between the included variables was assessed in this previous investigation and was not found for any of the variables.”

- Note that Table 1 does not appear to have a title

Thank you, the title has been added.

- Tables are difficult to read - suggest reformatting Tables 2 and 3 similarly to the supplemental tables as Cls are difficult to differentiate from ORs when not presented on separate lines

Table formatting appears to have been affected when the tables were inserted into the text. To correct, we have re-inserted the tables. We have also provided a separate file with the appropriately formatted tables to be used if needed.

- Suggest providing some discussion re: why no adjustments were made for potential clustering. It is anticipated that access to testing etc. would further differ between geographic regions within each province.

In this study we looked at neighbourhood-level characteristics and how they affect our outcomes of interest. While clustering to geographical areas is a very interesting question, it was outside the scope of our investigation, as we were interested in assessing province-wide phenomena and compare them to other provinces.

Results:

- Line 201 states that in all provinces individuals aged 20-34 and 35-49 were most likely to be tested, however

per the results presented in table 2, in NB all age groups appeared to have lower odds of being tested compared to 0-4 year olds

This has been revised to: “In Manitoba and Ontario, the 20-34 and 35-49 age groups were most likely to be

tested (Table 2, Figure 2), while children aged 0-4 showed the highest odds of being tested in New Brunswick.”

- Similarly, in line 206 it states that urban residents were more likely than rural residents to test positive in New Brunswick and Manitoba, however per Table 3 urban residents had higher odds of testing positive in NM and ON, not MB

This has been corrected, thank you.

- Per line 237, areas with higher ethnocultural composition exhibited lower testing rates (Table 2), however per Table 2 this is not universally true, i.e., for both MB and ON, odds of testing were higher in quintile 2 vs. 1 and quintile 3 vs. 1, and in quintile 4 vs. 1 for ON only.

This has been revised to “highest ethnocultural composition”.

- Suggest commenting on all significant findings, i.e., Table 2 shows that as situational vulnerability increased, there was decreased odds of being tested in MB (with the exception of those in the highest versus lowest vulnerability quintile), while the opposite was true in NB and ON.

As this is a very large data set with a lot of examined variables, we restricted our narrative analysis to those that show strong associations and clear trends. In the case of situational vulnerability and testing, the associations vary in direction across levels and the values are very close to 1, which in our opinion precludes the statement of strong consistent findings.

- Although not all variables were available for each province, e.g., asthma, air pollution, suggest commenting on these within the text where found to be significant rather than not commenting on these at all.

These variables were only provided in Supplemental data as additional information for researchers that may be interested in them. They were not included in the main body of the manuscript as we did not have a complete interprovincial data set for them, which precluded the assessment of trends across provinces. We would be happy to remove this additional data from Supplemental data if it is preferred.

Discussion:

- Consider providing more information regarding differences between provinces to assist in interpretation of results, i.e., was testing similarly available in each province (i.e., were individuals able to access testing in similar settings, or were there province-specific limitations), also were testing algorithms similar between provinces such that if each individual were to present in any of the 3 provinces would they have been tested? Were test availability and testing indicators consistent across the study period or were there any notable changes that may have influenced results? In ON, individuals working in LTC were required to submit to biweekly testing for a period of time, meaning these individuals may have been more likely to have a positive result identified - this may have influenced rates of testing in certain age groups, e.g. this may partially explain the finding in ON that those aged 20-34 and 35-49 had higher odds of testing relative to those aged 0-4.

We have added the following under Methods: “. In the early phases of the pandemic, access to testing was not widely available. Therefore, testing in all 3 provinces was limited largely to high-risk individuals, healthcare and personal care workers. For the majority of the time period of the study, all residents were eligible for SARS-CoV-2 testing in all provinces.

Despite earlier testing availability for the non-high-risk individuals as well as broader testing criteria, the Atlantic bubble likely worked to reduce infections in New Brunswick. We observed this effect both in reduced testing rates, as well as SARS-CoV-2 positivity, as compared to the other two provinces. This was addressed in the Discussion section.

- The results of this study are interesting - some additional discussion of findings would be helpful for the reader

Please see response to comments below.

- There is no discussion re: why children < 5 may have been more likely to be tested than all other age groups in

**NB**

On April 28, 2020, testing guidelines for children in New Brunswick were broadened to include more general symptomatic criteria, which may account for the observed high testing rates in the youngest age group in this province. New symptoms added in addition to previous ones (including fever above 38°C, a new cough or worsening chronic cough, sore throat, runny nose, headache) were:

- a new onset of fatigue;
- a new onset of muscle pain;
- diarrhea;
- loss of sense of taste or loss of sense of smell; and
- in children, purple markings on the fingers or toes. In the case of children with purple markings on their fingers or

toes, testing done even if none of the other symptoms are present.

We have added the following text to the Discussion section: “In addition, broader SARS-CoV-2 testing criteria for children than those in other provinces were introduced in New Brunswick in April, 2020, which we observed reflected in the high testing rate for the youngest demographic in this province.”

- Why might residential instability be associated with lower positivity?

A discussion of this finding has been added to the Discussion section, as follows:

“Interestingly, higher residential instability was found to be associated with lower odds of testing positive for COVID-19. The CIMD defines the index of residential instability at the national level as accounting for measures such as the proportion of the population who have moved in the past five years, the proportion of persons living alone, and the proportion of occupied units that are rented rather than owned. Therefore, it is a proxy for neighbourhood support and cohesiveness and household size. High levels of residential instability are likely to indicate a lower local community connection and a higher likelihood of living alone. Increased household size is, in turn, a well-established risk factor for COVID-19 infection.29 Therefore, our findings confirm that lower interpersonal opportunities of exposure, at the home or in the community, reduce the changes of COVID-19 positivity.

Reviewer #3: Please see overall comments and details to questions in the track changes in the attached document. The first page contains all information regarding major issues/concerns as well as minor issues. Within the document, the track changes provide more information.

These have been addressed within the tracked changes document.

While revising your submission, please upload your figure files to the Preflight Analysis and Conversion Engine (PACE) digital diagnostic tool, htt ps:/ / pacev2.apexcovantage.com/ . PACE helps ensure that figures meet PLOS requirements. To use PACE, you must first register as a user. Registration is free. Then, login and navigate to the UPLOAD tab, where you will find detailed instructions on how to use the tool. If you encounter any issues or have any questions when using PACE, please email PLOS at [figures@ plos.org.](mailto:figures@plos.org) Please note that Supporting Information files do not need this step.

https://ou tlook.office.com/mail/inbox/id/AAQkAGEzMGNhZGQwLTMwNmYtNDMyYy1 iMzY3LTUxNTFIYWQxZDNiZQAQANx%2B5mckxUFEmvxvOw. ..	5/5
